# Mutation Rules and the Evolution of Sparseness and Modularity in Biological Systems

**DOI:** 10.1371/journal.pone.0070444

**Published:** 2013-08-06

**Authors:** Tamar Friedlander, Avraham E. Mayo, Tsvi Tlusty, Uri Alon

**Affiliations:** 1 Department of Molecular Cell Biology, Weizmann Institute of Science, Rehovot, Israel; 2 Department of Physics of Complex Systems, Weizmann Institute of Science, Rehovot, Israel; 3 Simons Center for Systems Biology, Institute for Advanced Study, Princeton, New Jersey, United States of America; Hospital for Sick Children, Canada

## Abstract

Biological systems exhibit two structural features on many levels of organization: sparseness, in which only a small fraction of possible interactions between components actually occur; and modularity – the near decomposability of the system into modules with distinct functionality. Recent work suggests that modularity can evolve in a variety of circumstances, including goals that vary in time such that they share the same subgoals (modularly varying goals), or when connections are costly. Here, we studied the origin of modularity and sparseness focusing on the nature of the mutation process, rather than on connection cost or variations in the goal. We use simulations of evolution with different mutation rules. We found that commonly used sum-rule mutations, in which interactions are mutated by adding random numbers, do not lead to modularity or sparseness except for in special situations. In contrast, product-rule mutations in which interactions are mutated by multiplying by random numbers – a better model for the effects of biological mutations – led to sparseness naturally. When the goals of evolution are modular, in the sense that specific groups of inputs affect specific groups of outputs, product-rule mutations also lead to modular structure; sum-rule mutations do not. Product-rule mutations generate sparseness and modularity because they tend to reduce interactions, and to keep small interaction terms small.

## Introduction

Biological systems show certain structural features on many levels of organization. Two such features are sparseness and modularity [1]–[10]. Sparseness means that most possible interactions between pairs of components are not found. For example, less than 1% of the possible interactions are found in gene regulation networks of bacteria and yeast [11]. The second feature, modularity, is the near-decomposability of a system into modules - sets of components with many strong interactions within the set, and few significant interactions with other sets. Each module typically performs a specific biological function. Modularity is found for example in protein structure (functional domains) [12], in regulatory networks (gene modules, network motifs), and in body plans (organs, systems) - for reviews see [2], [8], [10], [13]. While modular networks are essentially sparse – sparse networks are not necessarily modular. Even if interactions are few, they could be evenly distributed and therefore not form modules.

Computer simulations of evolution are used to understand the origin of these structural features. The simulations begin with a set of structures, the elements of the structures are mutated, the fitness of each structure is evaluated according to a given goal, and then the structures with the highest fitness are selected. The most commonly used form of mutation in these simulations is the sum-rule mutation: adding a random number to the value of each element. Such simulations typically find optimal structures which satisfy the goal. However, they generally do not yield modular or sparse structures. Even when starting with a modular solution the simulations typically drift towards non-modular solutions, which are usually much more prevalent and are sometimes better at performing given the goal [14]. This leaves open the question of how and why sparseness and modularity evolve in biology.

Several studies have addressed this question by employing different approaches. For example, neutral models suggest that duplicating parts of a network can increase its modularity (“duplication-differentiation” model [15]) or similarly that mutation, duplication and genetic drift [16] can lead to modularity. Modularity in metabolic networks was suggested to arise from a neutral growth process [17], [18]. On the other hand, other studies suggest that modularity can be selected for, either indirectly or directly. Modularity has been suggested to be beneficial because it provides dynamical stability or robustness to recombination [19], improves the ability to accommodate beneficial foreign DNA [20], breaks developmental constraints [21], evolves due to selection for environmental robustness [22], [23] or because the same network supports multiple expression patterns [24]. Horizontal gene transfer, together with selection for novelty can lead to modularity in the polyketide synthase system [25]. It was recently suggested by Clune, Mouret and Lipson that network sparseness and modularity can evolve due to selection to minimize connection costs, as is thought to occur for example in neuron networks [26]. Kashtan *et al*. [27]–[29] found that when goals change with time, such that goals are made of the same set of subgoals in different combinations - a situation termed modularly varying goals (MVG) - the system can evolve modular structure. Each module in the evolved structure solves one of the subgoals, and modules are quickly rewired when the goal changes. Modularly varying goals tested in several model systems, with sum-rule mutations used when applicable [14], showed modularity under a range of parameters. Modularly varying goals also speed up evolution relative to unchanging goals [30], a phenomenon evaluated using analytically solvable models [14]. Due to the importance of sparseness and modularity in biology, it is of interest to see if additional mechanisms for their evolution exist. In particular, though attention has been given to the goals and cost functions, little attention has been given to the type of mutation rule used.

Here, we address the role of the mutation rule on the evolution of modularity and sparseness. Most studies that use simulations to study evolution employ a simple rule to specify how mutations change the parameters in the structure that is evolved - namely the ‘sum rule’, in which a parameter is mutated by adding a random number drawn from a specified distribution. Here, we note that this sum rule is usually not a good description of the effect of cumulative genetic mutations on a given biological parameter. Instead, the effects of mutations are better approximated by product-rule processes. For example, the effect of cumulative mutations on an enzyme’s activity is found to be multiplicative [31]. Similarly, the effect of mutation on binding of proteins to DNA [32], [33] and proteins to proteins [34]–[36] is thought to be multiplicative to a first approximation, such that the change in affinity caused by several genetic mutations is approximately the product of the effects of each mutation.

One fundamental reason for the use of product rule to describe the effect of genetic mutations is that mutations affect molecular interactions such as hydrogen bonds. This affects the free energy in an approximately additive way, assuming that the different molecular interactions are independent to a first approximation. Since affinity and reaction rate are exponential in free energy, the effects of cumulative genetic mutations on these parameters are approximately multiplicative. Note that in population genetics, there are different meanings to ‘additive’ and ‘multiplicative’ mutations [37], and thus we chose the terms ‘sum-rule’ and ‘product-rule’ to avoid confusion.

A related feature of mutations is that they more often reduce the absolute strength of the interaction or activity parameter than increase it [38]–[40]. This asymmetry can be captured using product-rule mutations: for example, multiplying by a random number normally distributed 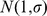 gives equal probability to multiply by 0.5 or 1.5, which tends to reduce the absolute size of the element; in order to revert a 0.5-mutation, one needs to multiply by a 2-mutation, which is less likely to occur.

To study the role of product-rule mutations, we compare evolution of simple and widely used model structures under sum-rule and product-rule mutations in computer evolution simulations. This is of interest because most simulations of evolution use sum-rules for mutations. We found that product-rule mutations lead to evolution of sparseness without compromising fitness. This relates to the study of Burda *et al*. which used a mutation rule that is approximately product-rule [41]. In contrast, we found that sum-rule mutations only lead to sparseness under special conditions, such as when the model parameters are constrained to be non-negative. Furthermore, when the goal is modular, we found that product-rule mutations led to modular structures, whereas sum-rule mutations generally do not. Unlike Kashtan *et al*., [14], [27], [28] here modularity arises from modular goals without need to change goals over time, and when there is no cost for connections. We study the speed and scaling laws of this process. The basic reason that product-rule mutations lead to sparseness and modularity is that they tend to reduce interaction terms and to keep small interaction terms small and thus cause the evolutionary dynamics to approach structures that have optimal fitness with minimal number of interactions. When goals are modular, this effect, in turn, leads to modular structure.

## Results

### A simple Matrix-multiplication Model of Transcription Networks

**Figure 1:**
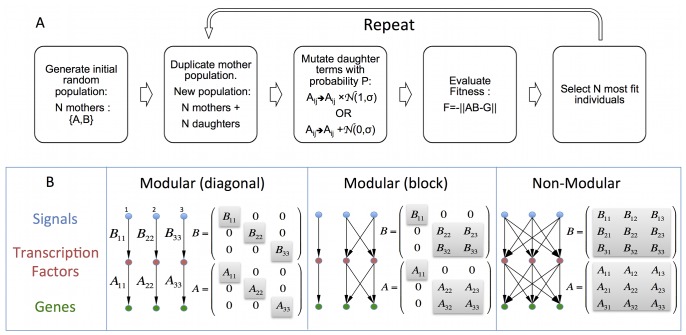
Evolutionary simulation scheme, and definition of model. (**A**) Simulation was initiated by randomly choosing 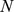 population members each consisting of 2 matrices 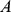 and 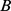. The next steps were repeated at each generation until the stopping condition was satisfied: the population was duplicated, one copy was kept unchanged and the other was mutated with probability 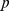. Mutation could be either sum-rule or product-rule. Fitness of all 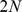 members (original and mutated) was evaluated by the distance of the product 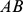 from a desired goal matrix 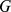, 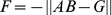, where 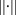 denotes the sum of squares of terms which is the square of 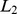 (Frobenius) norm. 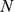 individuals were selected according to their fitness. Several selection methods were employed (see Text S1 for details). The simulation was stopped when the mean population fitness reached a value which was within a preset difference from the optimal fitness (usually 0.01). (**B**) Model represents a three layer network with a linear transformation function. Input signals 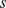 are transformed to intermediate layer activities (transcription factors) 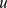 with 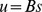. The intermediate layer is then transformed to output layer (gene expression) 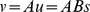. Modularity means block or diagonal structure of the matrices, corresponding to signals that affect only subsets of intermediate and output nodes.

To study the effect of the mutation rule on evolved structures, we use a standard evolutionary simulation framework [42], [43]. Briefly, the evolutionary simulation starts with a population of 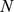 structures, duplicates them, and mutates each structure with some probability according to a mutation rule (the mutation rules described below will be our main focus). Fitness is evaluated for each structure in comparison to a goal. The fittest individuals are selected by a selection criterion, and the process is repeated, until high fitness evolves (Fig. 1A).

We consider, for simplicity, structures described by continuous-valued matrices. These serve as simple models for biological interactions, where the elements of the matrix 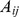 are the interaction strengths between components 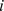 and 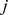 in the system. Evolution entails varying the matrix elements to reach defined goals. Linear matrix models have a long history in modeling of biological systems [41], [44]–[49]. Use of a matrix to describe gene expression is a standard approach. Several studies use matrices to reverse-engineer the underlying network [50]. Matrix models have also been used to understand developmental gene regulation, as in the pioneering work of Reinitz in Drosophila [51]–[53]; matrix models were recently used by De-Pace *et al.* to relate the strengths of regulation to the level of gene expression across fruit fly species using detailed gene expression measurements [54].

In the field of modularity, matrix models have been extensively used. Matrix models were used in the pioneering work of Lipson *et al*. [55] and also Wagner *et al*. [24]. We previously used a matrix model to analytically study a different route to modularity [14]. We evolved the matrix 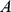 to satisfy the goal 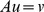, where 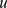 and 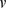 are vectors. The fitness is the distance to the goal, 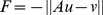 where 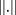 denotes sum of squares of elements (related to Fisher’s geometric model [56]).

Often, biological systems have multiple layers [57] where components in one level – e.g. receptors, send signals to components in the next level, e.g. transcription factors. We model this situation using a matrix multiplication model in which we evolve two matrices 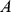 and 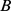 towards the goal 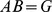, where 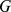 is a specified matrix that represents an evolutionary goal (Fig. 1B). The fitness in this case is 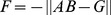. Note that there is an infinite number of matrix pairs 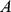 and 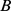 that satisfy a given goal 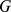.

As one concrete biological case, which may be kept in mind to guide the reader, the model can be interpreted in the context of a transcription network: if 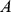 is the matrix connecting transcription factor (TF) activities to gene expression, the relationship 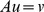 means that a vector of TF activities 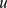 leads to a vector of gene expression 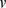. The matrix element 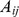 thus represents the regulatory strength of gene 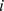 by TF 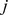. Similarly, if 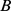 is a matrix of interactions between external signals 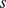 and TF activities, one finds that the TF activities are 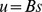. The matrix element 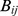 represents the effect of signal 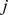 on TF 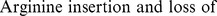. In total, the output gene expression vector that results from an input vector of signals 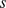 is 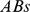. The goal 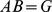 means that for every set of signals 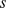, the gene expression at the output of the system is 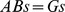, where 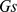 is the desired gene expression profile for input signals 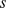 (see Fig. 1B).

### Product-rule Mutations Lead to Sparse Structures, Sum-rule Mutations do not

We compared sum and product mutation rules in evolving the model systems using an evolutionary simulation. The sum-rule is the commonly used addition of a normally distributed random number to a randomly chosen element of the matrices, which represents a mutation in the intensity of a single interaction between network components,
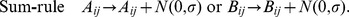



We also tested product-rules, in which an element of the matrix is multiplied by a random number. We tested
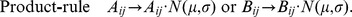



We study the case of 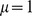, and also cases in which 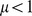 and 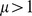. We also tested symmetric multiplication rules where the random number is log-normally distributed with 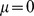 (see Text S1 for details), and thus has equal chance to increase or decrease the absolute strength of the interaction:



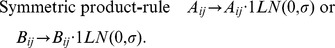
.

All cases gave qualitatively similar results, and most of the data below is for multiplying by 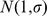. We also tested other forms of mutation distributions, including long tailed distributions that describe experimental data on sizes of mutation effects [Gamma distributions [39], see also [40], [58], [59] and references there], and found that the results are insensitive to the type of distribution used (see Movies S1, S2). Similarly, we tested the effect of mutation size, that is the parameter 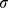, which we varied between 0.01 and 3, and found that the results are insensitive to this parameter. The evolutionary simulation and parameters are described in the Methods Section below.

**Figure 2:**
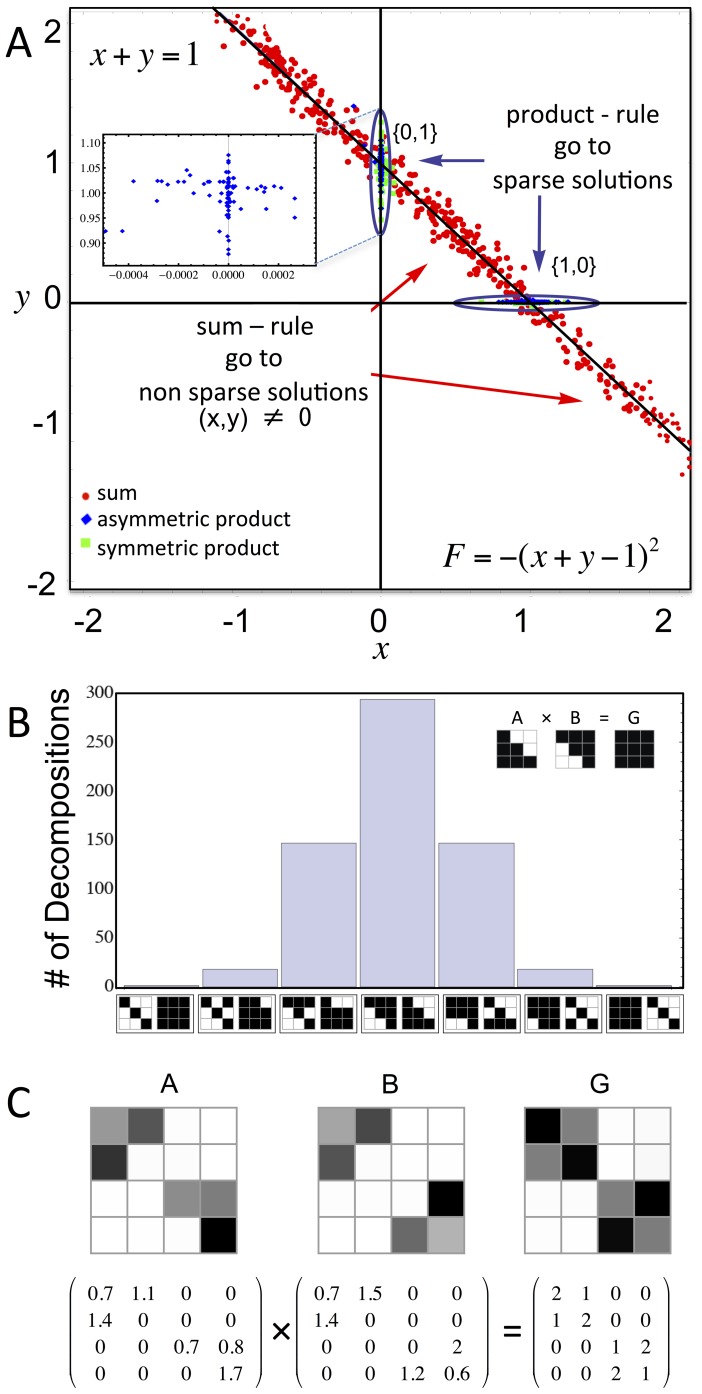
Product-rule mutations reach sparse solutions, sum-rule ones do not. (**A**) We demonstrate the difference between sum-rule and product-rule mutations in a simple 2–variable system 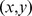, where the goal is that 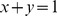. The optimal solutions lie on the line 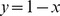. We compare solutions to this problem achieved by 3 different mutational schemes. Sum-rule mutations (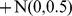 red circles) provide solutions that are spread along the line. In contrast, solutions achieved with both Gaussian product-rule (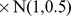 blue diamonds) and log-normal product-rule (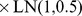 green squares) are concentrated near the intersection with the axes, i.e. near either (0,1) or (1,0). Since one coordinate is near zero, these are sparse solutions. Inset illustrates the solutions obtained with Gaussian product-rule mutations, demonstrating that matrix values can be negative as well as positive. Evolutionary simulation parameters were 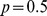, 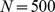, selection scheme was Boltzmann-like selection with 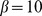. Simulations initiated utilizing random matrices with elements U(0,0.05). (**B**) Sparse solutions evolve in the matrix-multiplication model under product-rule mutations in response to a full-rank non-zero goal matrix 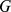. The solutions have the maximal number of zeros while still satisfying the goal. Zeros are distributed between the two matrices 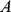 and 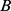. Shown are the possible configurations of 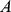 and 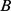 for matrices of dimension 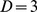, in which 6 zeros are distributed between the two matrices 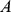 and 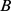. (**C**) In general, if the goal is block diagonal and full-rank, each of its blocks can be decomposed separately into blocks of 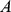 and 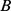, such that each block has the maximal number of zeros possible. Here we show an example in 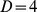, where 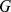 has 2 blocks of 2×2. The evolved 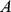 and 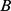 are such that each of their blocks is either an upper or a lower triangular matrix. Color represents numerical value (white = zero).

To demonstrate the effect of the mutation rule, we begin with a very simple model, namely a structure with two elements, 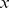 and 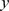, with fitness 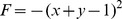. The optimal solutions lie on a line in the 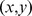 plane, namely 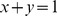 (Fig. 2A). Evolutionary simulations reach this line regardless of the mutation rule. Populations under sum-rule mutations evolve and spread out over the line. In contrast, product-rule mutations lead to solutions near the axes, either 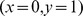, or 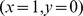. In other words, they lead to solutions in which one of the elements is close to zero – these are the sparsest solutions that satisfy the goal (see Fig. 2A, Movies S1, S2 and Figs. S10–S11 in Text S1).

The intuitive reason for the sparseness achieved by product-rule mutations is that once they are near a zero element, the size of the next mutation will be small (since it is a product of the element with a random number). Thus, the effective diffusion rate decreases (see Text S1). Strictly zero terms are fixed-points and near-zero terms remain small under mutations - so that the population becomes concentrated near zero elements. Sum-rule mutations, in contrast, show a constant drift rate regardless of the value of the elements. A full analytical solution of the dynamics of this simple model can be obtained by means of Fokker-Planck equations (see Text S1, Section 1), in excellent agreement with the simulations.

**Figure 3:**
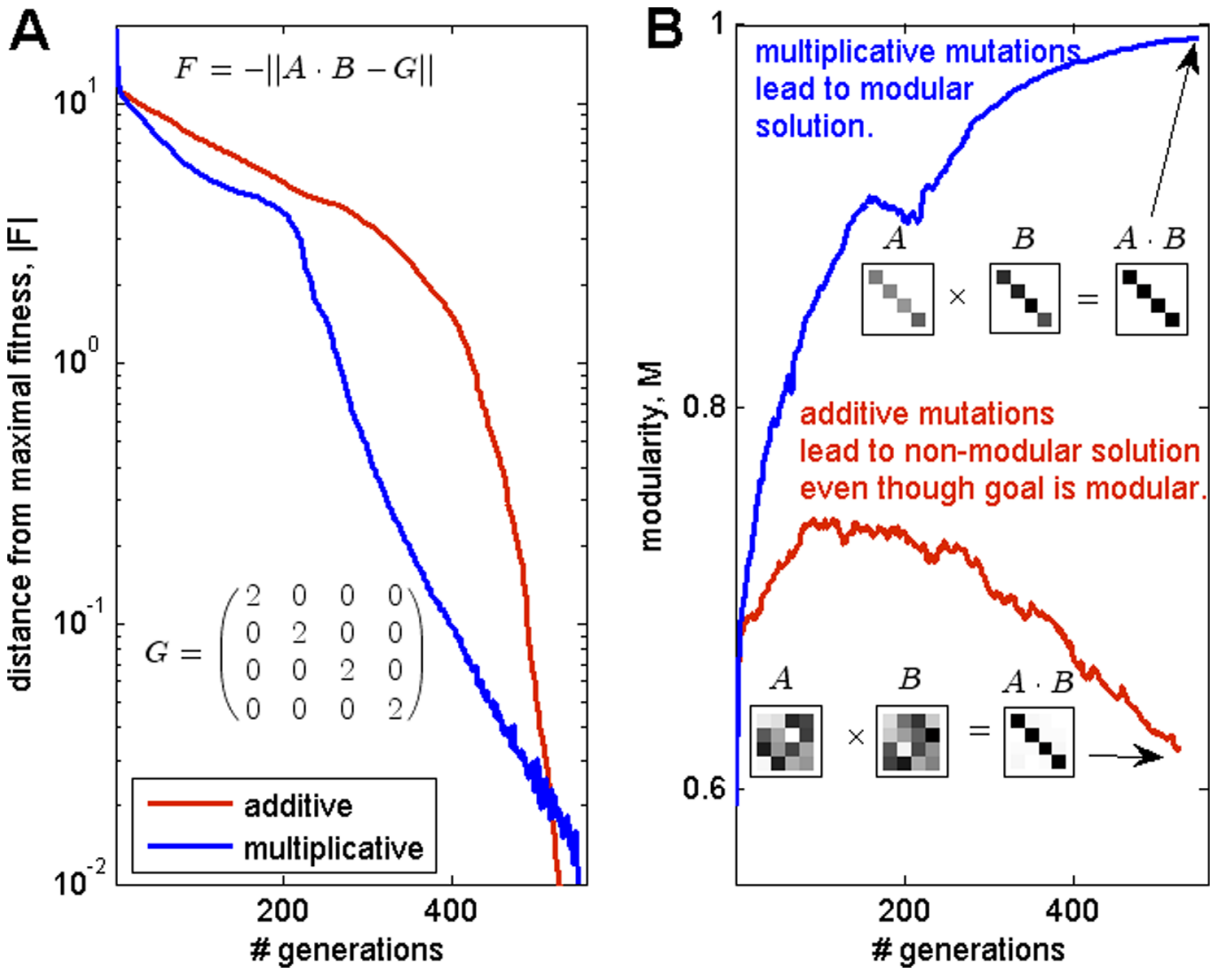
Product-rule mutations lead to modular structure under modular goal, sum-rule mutations do not. (**A**) Both sum-rule and product-rule mutations reach high fitness towards the goal 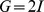. (**B**) Product-rule mutations reach high modularity, but sum-rule mutations do not. Simulations are in the matrix-multiplication model, matrix dimension 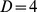. Examples of matrices drawn from the simulations are shown, with gray scale corresponding to element absolute value (white = zero). Fitness reaches a value of 0.01 due to constantly occurring mutations. Evolutionary simulation parameters are: sum-rule mutation size N(0,0.05), product-rule mutation size N(1,0.27), 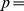 0.0031, tournament selection 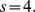
_._

We tested product-rule mutations also in the matrix-multiplication model, using as goals full rank matrices 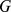. In numerical simulations, we refer to terms that are relatively small (<0.1% of the average element in 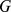) as “zero terms”, because strictly zero terms are not reached in finite time. We find that product-rule mutations lead to sparseness: matrices 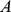 and 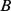 with the highest number of zeros possible while still satisfying the goal. In contrast, sum-rule mutations result in non-sparse solutions 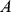 and 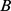 with non-zero elements (Fig. 3).

The sparse solutions found with product-rule mutations have many zero terms, whose number can be computed by means of the LU decomposition theorem of linear algebra. The LU decomposition expresses a nonsingular matrix as a product of an upper triangular matrix and a lower triangular matrix [60]). The total number of zeros in 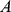 and 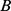 is the number of zero elements in the LU decomposition of 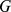. This number can be calculated exactly: for a given full rank matrix 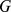 of dimension 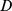 with no zero elements, the maximal number of zeros in 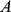 and 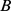 together is 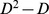 (for proof see Text S1). This result is found in our simulations.

The zeros are distributed between 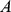 and 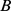 in various ways in the different simulations: Sometimes 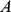 and 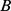 are both (upper and lower) triangular, each with 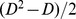 zero elements. Other runs show one full matrix with no zeros and the other a diagonal matrix with 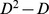 zeros. All other distributions of zeros are also found (Fig. 2B–C; Fig. S16 in Text S1 for comparison with sum-mutations). When 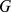 is full rank and has 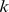 zeros, the total number of zeros in the evolved matrices 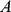 and 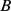 is 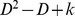, again the maximal possible number of zeros in matrices that show optimal fitness (for proof see Text S1).

We note that there is a special situation in which sum-rule mutations can also lead to sparseness in the present models. This occurs when the models are constrained to have only non-negative terms 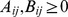. In this case, the sum rule, constrained to keep terms non-negative – for example, by using 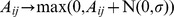, can also lead to sparseness. This relates to known results from non-negative matrix factorization [61]. However, in general biological models, structural terms are expected to be both negative and positive, representing, for example, inhibition and activation interactions between components. Our mechanism for the evolution of sparseness and modularity is different from non-negative matrix factorization and works regardless of the sign of the interaction terms (see for examples Fig. S15 in Text S1 and Movies S1, S2).

### When the Goal is Modular, Product-rule Mutations Lead to Modular Structure; Sum-rule Mutations do not

Up to now, we considered goals 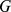 which are described by general matrices. We next limit ourselves to the case where the goals 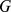 are described by matrices which are modular, for example, diagonal or block diagonal matrices. The main result is that when the goals are modular, the evolved structures 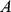 and 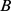 are also modular if mutations are product-rule; in contrast, sum-rule mutations lead to 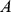 and 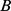 that are not modular despite the fact that the goal is modular.

We first define modular structures and modular goals in the context of the present study. Modular structures are structures that can be decomposed into sets of components, where each set shows strong interactions within the set and weak interactions with other sets [1], [2], [10], [62] (Fig. 1B). Here, modular structure means block-diagonal matrices. For ease of presentation, we first consider the most modular of structures – namely diagonal matrices. We define modularity by 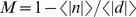 where 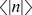 and 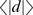 are the mean absolute value of the non-diagonal and diagonal terms respectively, and where we permute rows/columns to maximize modularity 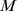 (same permutation for rows of 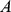 and columns of 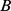, see Text S1). Thus, a diagonal matrix has 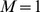, and a matrix with diagonal and non-diagonal terms of similar size has 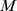 close to zero.

Modular goals are goals which can be satisfied by a modular structure. Modular goals in the present models are represented by diagonal or block-diagonal goal matrices 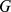. These goals, in the biological interpretation of transcription networks (Fig. 1B), are goals in which each small set of signals affects a distinct set of genes, and not the rest of the genes. For example, the signal lactose affects the *lac* genes in *E. coli*, whereas a DNA damage signal affects the *SOS* DNA-repair genes, with little crosstalk between these sets. Other examples for biological goals that are modular are sugar metabolism [63] and the tasks of chemotaxis and organism development (see detailed discussion in [27]). All are composed of several sub-tasks that are associated with different sets of genes.

We note that a modular goal does not necessarily lead to modular structures. For example the goal 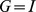 is modular since 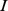 is the diagonal identity matrix. This modular goal can be satisfied by a product of infinitely many pairs of non-modular matrices 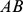. In fact, for every invertible 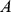, the inverse 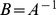 satisfies the goal. As a result, the vast majority of the possible solutions are non-modular (modular solutions have measure zero among possible solutions to 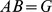). In line with this observation, we find that simulations with sum-rule mutations lead to solutions with optimal fitness (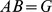), but with non-modular structure 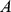 and 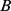 (Fig. 3, Fig. S16 in Text S1).

In contrast, we find that product-rule mutations lead to modular structures 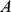 and 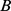, for a wide range of parameters. For the goal 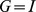, the evolved 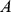 and 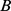 are both diagonal matrices, with elements on the diagonal of 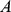 that are the inverse of the corresponding elements on the diagonal of 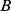. Thus 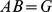. Similar results are found if the goal is nearly modular (e.g. diagonal with small but nonzero off-diagonal terms): in this case, the evolved 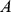 and 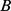 are both nearly diagonal (Fig. S14 in Text S1).

We also studied block-modular goals. In this case, product-rule mutations led to block-modular matrices 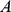 and 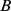, with the same block structure as the goal matrix 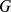 (Fig. 2C). Each of the blocks in the matrices 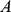 and 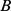 had the maximal number of zeros possible so that the product of the two blocks is equal to the corresponding block in the goal matrix 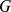 (the total number of zeros is equal to that in the LU decomposition of each block) – compared to Fig. S16 in Text S1 (block-diagonal goal with sum-rule mutations).

It is important to note that in order to observe the evolution of modularity in the present setting, the selection criteria should not be too strict, otherwise non-modular solutions cannot be escaped effectively (Text S1). In other words, overly strict selection does not allow the search in parameter space needed for product-rule mutations to reach near-zero elements. In the present simulations, we find evolution of modularity using standard selection methods including tournament, elite (truncation) and continuous Boltzmann-like selection (see Methods, and Text S1 for analysis of sensitivity to parameters).

### Time to Evolve Modular Structure Increases Polynomially with Matrix Dimension

**Figure 4:**
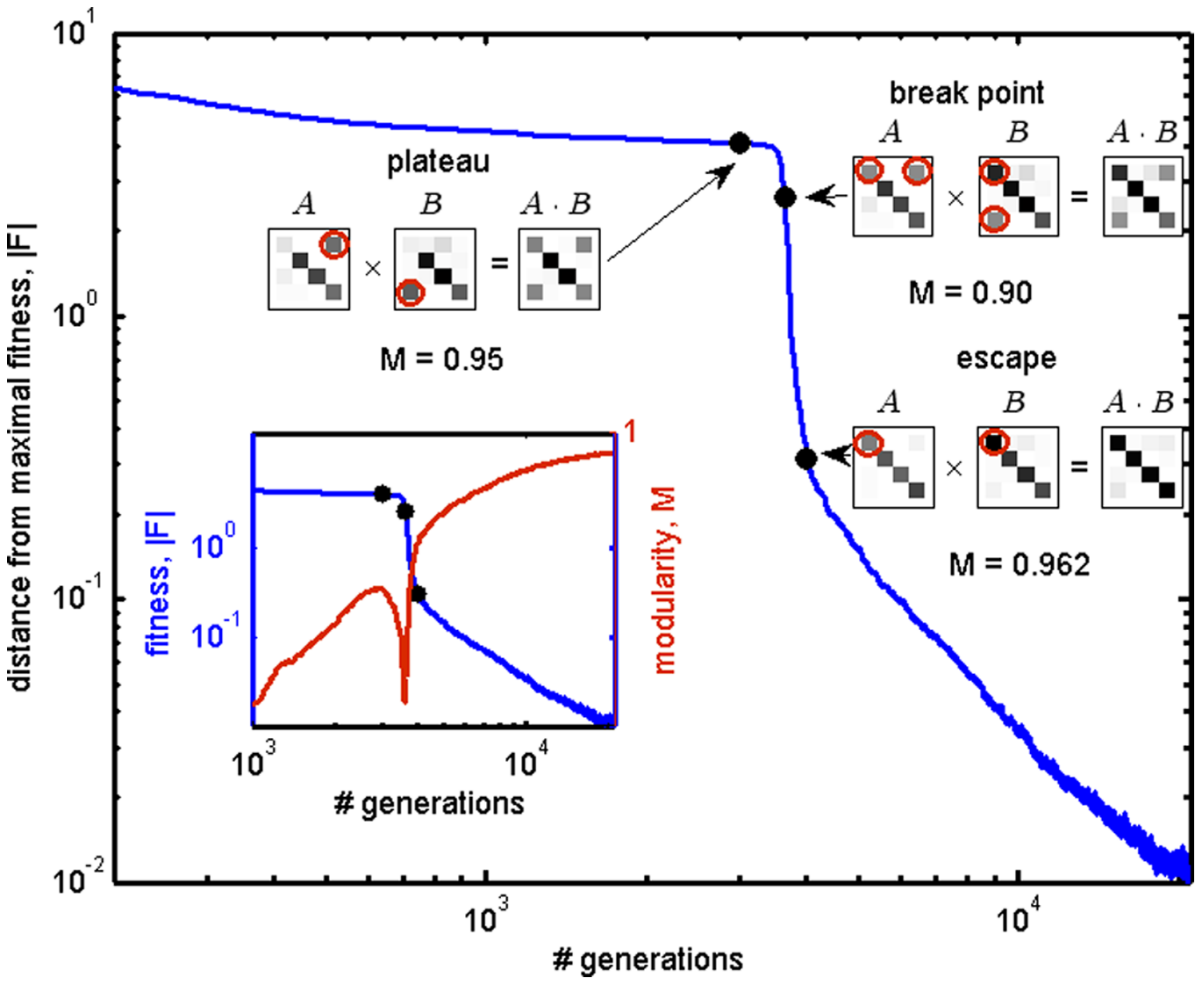
Escape from a fitness plateau entails a temporary decrease in modularity. Mean distance from maximal fitness as a function of time in the matrix-multiplication model with product-rule mutations, towards a diagonal goal. Note the plateau in the dynamics. Matrices and their modularity drawn from the simulations at different time-points (designated by black points) are shown, with gray scale corresponding to element absolute value (white = zero). Inset: mean modularity of population (red curve), showing a sharp decrease at the time of escape from the plateau (same time points are shown). In order to escape the plateau (“break point”), the circled terms in 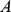 and 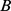 are changed. This occurs through a simultaneous increase of the new term and decrease of the old one, such that temporarily modularity is decreased (see inset). Finally, the correct arrangement of terms is attained (“escape”) and modularity increases again. Fitness reaches a value of 0.01 due to constantly occurring mutations.

We studied the dynamics of the evolutionary process in our simulations with product-rule mutations. We found that over time, fitness and modularity both generally increase, until a solution with optimal fitness and maximal modularity is achieved. We found that the matrix multiplication model often shows plateaus where fitness is nearly constant, followed by a series of events in which fitness improves sharply (Fig. 4) [22], [64]. In these events, modularity often drops momentarily. Analysis showed that the plateaus represent non-modular and sub-optimal structures. A mutation occurs which reduces modularity but allows the system to readjust towards higher fitness, and then to regenerate modularity.

**Figure 5:**
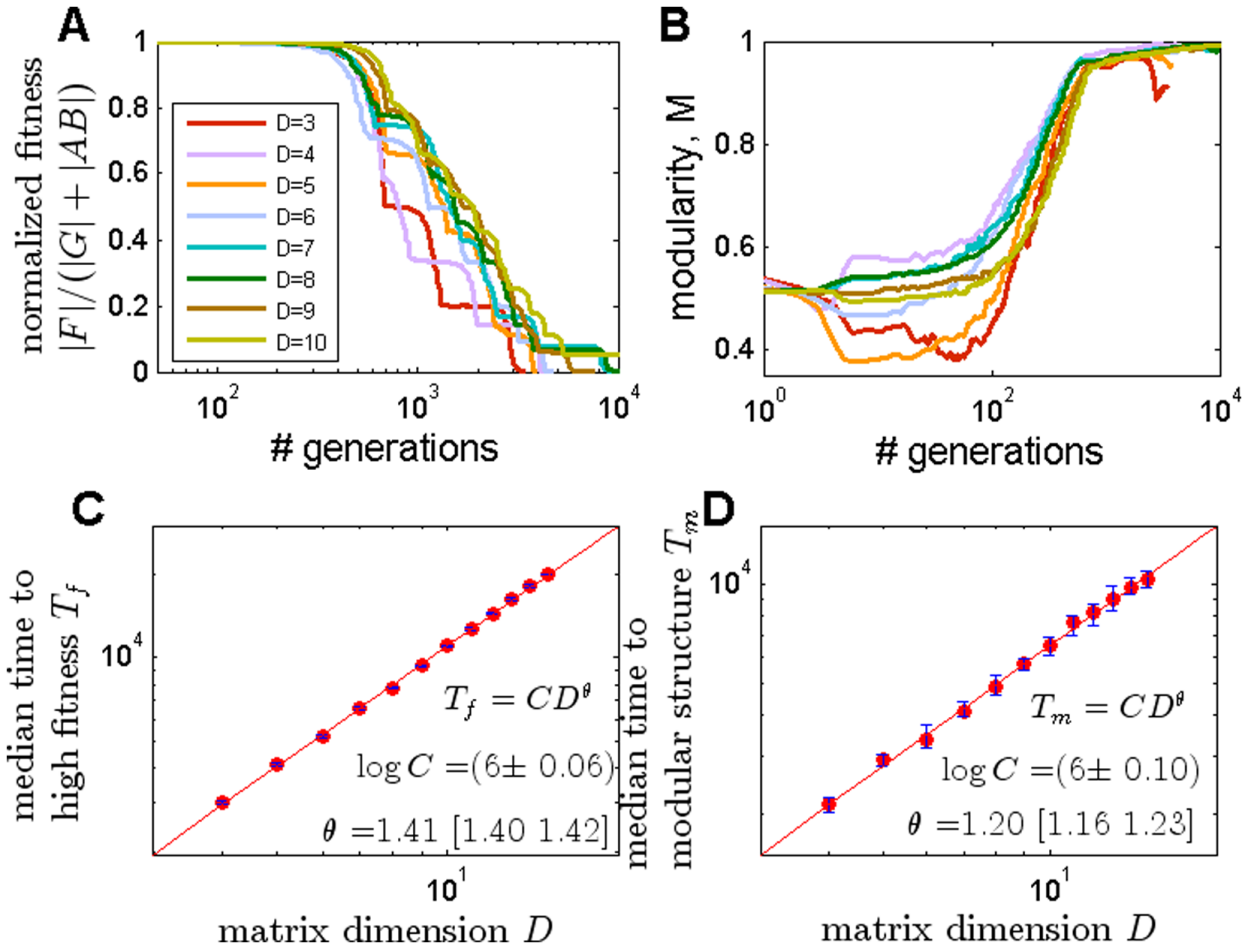
Time to high fitness and modularity grows polynomially with dimension. (**A**) Normalized distance to maximal fitness 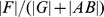 as a function of generations in the matrix-multiplication model evolved towards 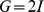, for matrix dimensions 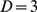 to 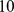. Each color represents a different value of 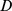. Curves typically have 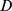 “steps”, where each step corresponds to the build-up of an additional significant term. (**B**) Modularity in the same simulations. (**C**) Median time (generations) to high fitness (distance from maximal fitness <0.01) as a function of the dimensions of the matrices 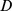 goes as 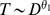 with 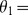
1.41 [1.40, 1.42] [CI 5%, 95%]. (**D**) Median time (generations) to modular structure (see methods for dimension dependent criterion for high modularity) goes as 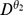 with 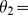
1.20 [1.16, 1.23]. Initial conditions are random matrices with small elements drawn from U(0,0.1). Element-wise mutation rate 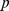 at all simulations was 0.0005; product-rule mutations normally distributed N(1,0.1) See Text S1 for details on error calculation in **C**–**D**.

We also tested the time to reach high fitness solutions, and its dependence on the dimension of the matrices 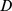. The time to high fitness solutions depends on the settings of the simulations: initial conditions, selection criteria and mutation rates and size, and the stopping criteria of the simulations. Here we present results in which time to high fitness was measured as the median time over repeat simulations to reach within 0.01 of optimal fitness, with product mutation rule 
N(1,0.1) and probability of mutation per element that is dimension-independent (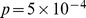). Initial conditions were matrices with small random elements (U(0,0.05)). The time to high fitness increased approximately as 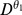 with 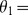
1.40+/−0.01 and the time to modularity (see Methods for definition) increased as 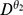 with 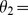
1.21+/−0.04 (Fig. 5).

## Discussion

We found that product-rule mutations lead evolution towards structures with the minimal number of interaction terms that still satisfy the fitness objective. Thus, product-rule mutations lead to sparseness. When the goal is modular, product-rule mutations lead to modular structure. This is in contrast to sum-rule mutations, which lead, under the same conditions, to non-sparse and non-modular solutions.

The mechanism by which product-rule mutations lead to sparseness and modularity is that near-zero interaction terms are kept small by product-rule (but not sum-rule) mutations. A second effect is mutation asymmetry, where it is more likely to reduce an interaction than increase it. However, using a symmetric product-rule (multiplying by a number drawn from a symmetric log-normal distribution) combined with selection still leads to sparseness and modularity, because selection also breaks the symmetry. Once a parameter becomes small, product rule mutations keep it small (as opposed to sum-rule mutations). This creates a dynamic ‘trap’ in which the steady state distribution of phenotypes is highly enriched with near zero parameters. Thus, the mutational asymmetry effect is not essential for the present conclusions (see Figs. S10–S11 in Text S1 and Section 2 in Text S1). Furthermore, in special situations a sum-rule can also lead to sparseness, namely if the structural terms in the model are constrained to be non-negative. We note that sparseness can also be enhanced in some networks due to physical constraints such as spatial/geometric limitations in networks that describe protein structure [12] or neuron wiring networks [65].

We used a simple but general model of biological systems, namely linear matrix models, and matrix multiplication models. These models have been widely used to describe gene regulation, neuronal networks, signal transduction and other systems [41], [44]–[49], [66], [67]. The matrix multiplication model is a commonly used model for three layer systems, such as signals 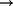 transcription factors 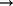 genes. As in many biological models, many combinations of parameters can achieve the same goal.

We believe that the present mechanism has generality beyond the particular model used here. Consider a general map 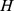 between a coarse-grained genotype 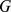 (described as a set of biochemical parameters and interaction parameters) and phenotype 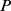, 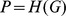. The optimal phenotype 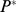 is obtained by a manifold of different genotypes 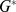. Given reasonably strong selection relative to genetic drift and mutation, evolutionary dynamics will reach close to this manifold. One can then ask how the mutation rule affects evolutionary dynamics along this manifold. Sum-rule mutations lead to a random walk on the manifold that does not prefer regions with small parameters, whereas product-rule mutations lead to solutions with the maximal number of zero (very small) parameters: once evolution comes close to a zero parameter, product-rule mutations keep that parameter small.

Product-rule is a more realistic description of the effect of cumulative genetic mutations on a biochemical parameter than sum-rule mutations, because of the nature of biological interactions. The effect of genetic mutations was also shown in several experimental studies to be asymmetric (for example [38]–[40]), with bias to decrease interactions, enzymatic activity [38] or body size [40]. The discussion of symmetric product rule mutations (that is - multiplying by log-normally distributed random numbers) is given here for completeness, and not because of biological relevance. Further studies can use other microscopic models for mutations (such as Ising-like models for bonds between macromolecules [41], [68]), and explore the effect of mutations that set interactions to near-zero with large probability. Due to the inherent product-rule nature of biological mutations, we could not think of experimental tests that can compare sum-rule to product-rule mutations, beyond computer simulations or experiments in the realm of electronics [69], [70] or mechanics [71].

The present mechanism does not exclude previous mechanisms for the evolution of modularity. In fact, it can work together with other mechanisms and enhance them. For example, in Kashtan *et al*. [14], [27], [29], [30], modularity evolved when the modular goal changed over time (MVG mechanism). In the present study, no change of the goal over time is required. Using product-rule mutations in the models of Kashtan *et al*. (instead of the original sum-rule mutations) is expected to enhance the range of parameters over which modularity evolves. Supporting evidence was recently provided by Clune *et al*. [26] that demonstrated how a different mechanism for the evolution of sparseness significantly enlarges the range of parameters over which the MVG mechanism produces modular structures. Another difference from some previous studies is that modularity evolves here with no need for an explicit cost for interaction terms in the fitness function [14], [27], [29], [30], [72]. Adding such a cost, as in Clune *et al*., [26] would likely enhance the evolution of sparseness and modularity. It would be intriguing to search for additional classes of mechanisms to understand the evolution of sparseness, modularity, and other generic features of biological organization [73].

## Materials and Methods

### Evolutionary Simulation

Simulation was written in Matlab using standard framework [42], [43]. All source codes, data and analysis scripts are freely available in a permanent online archive at http://dx.doi.org/doi:10.5061/dryad.75180. We initialized the population of matrix pairs by drawing their 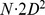 terms from a uniform distribution. Population size was set as *N* = 500. In each generation the population was duplicated. One of the copies was kept unchanged, and elements of the other copy had a probability *p* to be mutated – as we explain below. Fitness of all 2*N* individuals was evaluated by 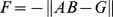, where 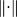 denotes the sum of squares of elements [56]. The best possible fitness is zero, achieved if *AB* = *G* exactly. Otherwise, fitness values are negative. In the figures we show the absolute value of mean population fitness, which is the distance from maximal fitness (Fig. 3–4, Fig. S12 in Text S1), or the normalized fitness 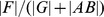 (Fig. 5). The goal matrix was either diagonal , nearly-diagonal (diagonal matrix with small non-diagonal terms), block-diagonal or full rank with no zero elements. 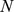 individuals are selected out of the 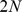 population of original and mutated ones, based on their fitness (see below). This mutation–selection process was repeated until the simulation stopping condition was satisfied (usually when mean population fitness was within 0.01 of the optimum).

#### Mutation

We mutated individual elements in the matrix. We set mutation rate such that on average 10% of the population members were mutated at each generation, so the element-wise mutation rate was This relatively low mutation rate enables beneficial mutants to reproduce on average at least 10 generations before they are mutated again. In simulations where we compared dependence on matrix dimension (Fig. 5) we used the same mutation rate at all dimensions, generally the one that pertains to the highest dimension used in the simulation.

We randomly picked the matrix elements (in both 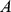 and 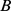) to be mutated. Mutation values were drawn from a Gaussian distribution (unless otherwise stated). For sum-rule mutations, this random number was added to the mutated matrix value: 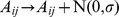 or 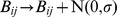, and for product-rule mutation, the mutated matrix element was multiplied by the random number: or 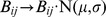. Mean mutation value 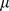 was usually taken as 1, however we also tested other values of 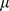 (both larger and smaller than 1) and other mutation distributions (Gamma and log-normal) and results remained qualitatively similar, although the time-scales changed. In most simulations shown here we used 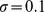 (unless stated otherwise). Fitness convergence and its time scale depend on the mutation frequency and size, as demonstrated in our sensitivity test (Text S1).

#### Selection methods

We tested 3 different selection methods and all gave qualitatively very similar results with only a difference in time scales. Most results presented here were obtained with tournament selection with group size S = 4 (see [43] chap. 9). We also tested truncation-selection (elite) [42] and proportionate reproduction with Boltzmann-like scaling [41], [55], [74]. For a detailed description see Text S1.

#### Definition of modularity

If the goal is diagonal, we define modularity as 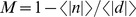 where 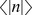 and 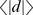 are the mean absolute value of the non-diagonal and diagonal terms respectively. At each generation, the 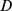 largest elements of each matrix (both 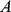 and 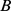), were considered as the diagonal 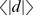 and the rest 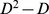 terms as the non-diagonal ones 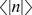. Averages were taken over matrix elements and over the population. This technique copes with the unknown location of the dominant terms in the matrices, which could form any permutation of a diagonal matrix. Thus, 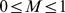: a diagonal matrix has 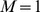, and a matrix whose terms are all equal has 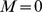. Since we choose the largest elements to form the diagonal, negative values of 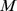 do not occur. When the goal is non-diagonal, one can use standard measures for modularity such as (49) [not used in the present study].

#### Calculation of time to modular structure

To estimate the time when modular structure is first obtained, we used the following approximation for fitness value with diagonal goal. Assume that 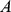 and 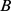 are 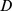
- dimensional matrices consisting of 2 types of terms: diagonal terms all with size 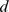 and non-diagonal terms all with size 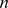 and that the goal is 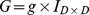. The fitness then equals:
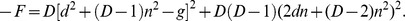



We collect terms by powers of 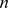, and obtain a constant term and terms with powers 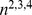. Modular structure is obtained when the solution has the correct number of dominant terms at the right location and their size is approximately 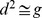. At the beginning of the temporal trajectory, when non-diagonal elements are relatively large, 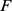 is dominated by the 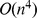 term. When a modular structure emerges, non-diagonal elements become relatively small, and the dominant term remaining in 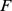 is 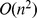. Our criterion for determining time to modular structure was the time when the 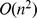 term first became dominant, i.e. when 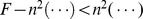.

#### Supplementary movies

Movies demonstrate the simulation dynamics in the problem under product-rule mutations with various distributions of mutations. All distributions converge to either of the sparse solutions.

## Supporting Information

Text S1 **Contains the following additional data:** 1. Analytical solution and simulations of toy model, 2; 2. Mutation properties: product vs. sum mutation, mutation symmetry, 11; 3. Evolutionary simulations – detailed dexcription, 16; 4. Evolutionary simulation parameter sensitivity test, 18; 5. Modularity: definitions and error calculation, 20; 6. LU decomposition – proofs, 21; 7. Nearly modular - supplementary figure, 23; 8. Mutation sign and distribution – supplementary figure, 24; 9. Block diagonal goal – supplementary figure, 25.
(PDF)

Movie S1 Mutations had Gamma distribution with parameters Gamma(1, 40.25). In addition, each mutation value was multiplied by −1 with probability 0.1, so that matrix values could also change their sign. - selection was used with.
(AVI)

Movie S2 Mutations had log-normal distribution with parameters LN(−0.11, 0.47). In addition, each mutation value was multiplied by −1 with probability 0.1, so that matrix values could also change their sign. - selection was used with.
(AVI)
