# Correction: Mutation Rules and the Evolution of Sparseness and Modularity in Biological Systems

**DOI:** 10.1371/journal.pone.0118129

**Published:** 2015-03-06

**Authors:** The PLOS ONE Staff

In the Results, under “Product-rule Mutations Lead to Sparse Structures, Sum-rule Mutations do not”, the Symmetric product-rule should read as follows:

$$A_{ij}\to A_{ij}\cdot \text{LN}(0,\sigma )\;\text{or}\;B_{ij}\to B_{ij}\cdot \text{LN}(0,\sigma )$$
