# Supplementary material for: Mutation Rules and the Evolution of Sparseness and Modularity in Biological Systems
Source: PLoS One. 2013 Aug 6;8(8):e70444. doi: 10.1371/journal.pone.0070444 (PMC3735639; doi:10.1371/journal.pone.0070444)
Supplement: Text S1 — Contains the following additional data: 1. Analytical solution and simulations of toy model, 2; 2. Mutation properties: product vs. sum mutation, mutation symmetry, 11; 3. Evolutionary simulations – detailed dexcription, 16; 4. Evolutionary simulation parameter sensitivity test, 18; 5. Modularity: definitions and error calculation, 20; 6. LU decomposition – proofs, 21; 7. Nearly modular - supplementary figure, 23; 8. Mutation sign and distribution – supplementary figure, 24; 9. Block diagonal goal – supplementary figure, 25. (PDF) [file pone.0070444.s001.pdf]

Mutation Rules and the Evolution of Sparseness and Modularity in Biological Systems

Tamar Friedlander, Avraham E. Mayo, Tsvi Tlusty and Uri Alon

Supporting Information

Table of Contents

1. Analytical solution and simulations of toy model 2
2. Mutation properties 11
3. Evolutionary simulations 16
4. Evolutionary simulation parameter sensitivity test 18
5. Modularity: definitions and error calculation 20
6. LU decomposition - proofs 21
7. Nearly modular $\boldsymbol{G}$ - supplementary figure 23
8. Mutation sign and distribution – supplementary figure 24
9. Block diagonal goal – supplementary figure 25

1. Analytical solution and simulations of toy model

To gain better insight into the effect of the product mutational mechanism we studied a simple toy model. We showed that the effect of sum-mutations is equivalent to free diffusion in isotropic medium along equal fitness lines with no preference to any specific solution on this line. The effect of product-mutations in contrast is described by diffusion in the log-transformed parameter domain. In the original domain the population is log-normally distributed and asymptotically approaches zero. If selection were absent this mutational mechanism would nullify all network interactions. However the combination of the product-mutations with selection for achieving a certain goal results in solutions with maximal number of zeros that still satisfy the goal. The dynamics and type of solutions demonstrated in this model is representative of those we obtained in simulations of the more complex matrix-multiplication model described in the main text.

We study the simplest model in which there is an excess degree of freedom, namely a two variable model such that a modular solution is enabled. We assume that the fitness function depends on the two variables only through their sum. That is, the population exists in a 2-variable space (x, y) and its goal is to reach the line where $\mathrm{x} + \mathrm{y} = 1$. All points on this line are equally fit, but only two of them - the intersections with the axes (0,1) and (1,0) are sparse. This is because we interpret the variables as interaction intensities between network components and a sparse network is one in which some interactions are zero. Fitness is evaluated by the square distance from this line $\mathrm{F}(\mathrm{x}, \mathrm{y}) = -(\mathrm{x} + \mathrm{y} - 1)^2$. Although the model does not include terms which depend on products of variables (as in the more general model that we simulated) it is still useful for comparing the effects of the sum and product mutational schemes on the evolutionary dynamics.

In our analysis of the toy model we made a number of simplifying assumptions with respect to the simulations. First we assume continuous time, instead of the discrete generations in the evolutionary simulation. We also take the limit of infinitely large mutation rate with infinitesimally small mutation size, such that their product is finite, and can be described by a diffusion coefficient (compare to [1]). Furthermore we take the limit in which population size is very large, so that fluctuations and random drift due to finite size effect are negligible.

The population dynamics is naturally decomposed into two axes: along equal-fitness lines dynamics is mutation (diffusion) dominated; in perpendicular to such lines it is determined by a combination of mutation and selection (see Fig. S1). We solve analytically the dynamics of the diffusion-dominated axis, and quantify the speed with which sparse solutions are approached with product-mutations. We also obtain a steady-state solution for the mutation-selection axis, showing that it obeys a Boltzmann distribution. We demonstrate our findings by detailed stochastic simulations (see below), showing good agreement with the analytical solutions.

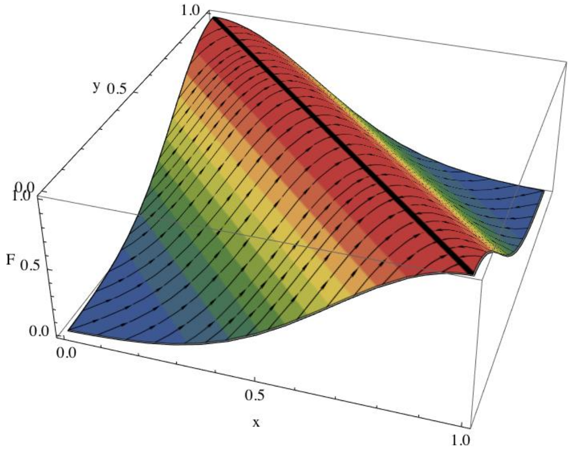

Fig S1: Decomposition of the $\mathbf{x}, \mathbf{y}$ problem into its natural axes. Color represents fitness. Along equal fitness lines only mutation (diffusion) plays a role. Arrows show the other axis along which both selection and mutation are active. The maximal fitness line $\mathbf{x}+\mathbf{y}=\mathbf{1}$ is shown in bold.

Dynamics under sum-rule mutation

The mutation-selection dynamics of the population is approximately captured by the Fokker-Planck (abbreviated below 'FP') equation [2]. We denote by $\mathrm{P}(\mathrm{x},\mathrm{y},\mathrm{t})$ the population distribution at time t. It is subject to the potential $\mathrm{F}(\mathrm{x},\mathrm{y}) = -(\mathrm{x}+\mathrm{y}-1)^2$ (selection), and diffusion with coefficient D (mutation):

$$\partial_{\mathrm{t}}\mathrm{P}(\mathrm{x},\mathrm{y},\mathrm{t}) = \mathrm{D}\,\nabla^2\mathrm{P}(\mathrm{x},\mathrm{y},\mathrm{t}) - \nabla\cdot\big(\mathrm{P}(\mathrm{x},\mathrm{y},\mathrm{t})\nabla\mathrm{F}(\mathrm{x},\mathrm{y})\big).$$

The Fokker-Planck equation is the continuous second order approximation of the more general master equation describing the dynamics of a population subject to probabilistic transitions between states. For example in the 1-dimensional case the master equation takes the form:

$$P(x,t+\Delta t) = P(x,t) + \sum_z w(z,\mathrm{x})\mathrm{P}(\mathrm{z},\mathrm{t}) - \sum_z w(x,\mathrm{z})\mathrm{P}(\mathrm{x},\mathrm{t})$$

where $w(z,x)$ is the transition probability from z to x. By second order approximation we neglect transitions between grid points which are far from one another (i.e., mutations generally have a small effect). This assumption translates to w being a narrow function of its arguments and is common practice in the literature (see for example [3]). We also assume that the transition probability $w(z,x)$ depends only on the difference in fitness between x and z. For example $w(z,\mathrm{x}) = e^{(F(z)-F(x))}$. With this we obtain:

$$P(x,t+\Delta t) = P(x,t) + e^{(F(x+\Delta\mathrm{x})-F(x))}\mathrm{P}(x+\Delta\mathrm{x},\mathrm{t}) + e^{(F(x-\Delta\mathrm{x})-F(x))}\mathrm{P}(x-\Delta\mathrm{x},\mathrm{t}) - \left(e^{(F(x)-F(x+\Delta\mathrm{x}))} + e^{(F(x)-F(x-\Delta\mathrm{x}))}\right)\mathrm{P}(\mathrm{x},\mathrm{t}).$$

Expanding this equation to first order in Δt and second order in Δx, and rearranging we obtain the Fokker-Planck type equation as above with the diffusion coefficient D given by $\Delta x^2/\Delta t$ as usual [3].

Taking into account the specific form of the fitness function F in this problem, it is convenient to make the following coordinate transformation:

$$u = x + y$$

$$v = x - y$$

With that, the potential and the FP equation transform to:

$$\tilde{F}(u) = -(u-1)^2$$

$$\partial_t \tilde{P}(u,v,t) = 2D\,\partial_u\,\partial_u \tilde{P} + 2D\,\partial_v\,\partial_v \tilde{P} - \partial_u \tilde{F}\,\partial_u \tilde{P} - \tilde{P}\,\partial_u\,\partial_u \tilde{F}.$$

This equation can be solved by separation of variables. We assume that the population already converged to the line of optimal fitness, $u = 1$. Therefore the time dependence of P enters only through $\tilde{P}(u,v,t) = V(v,t)U(u)$. The equation then reads:

$$\frac{\partial_t V - 2D\,\partial_v\,\partial_v V}{V} = \frac{2D\,\partial_u\,\partial_u U - \partial_u \tilde{F}\,\partial_u U - U\,\partial_u\,\partial_u \tilde{F}}{U} = \alpha = \text{const.}$$

For $\alpha \neq 0$ the equation in V describes a population growing at rate α. In our simulations we keep the population size constant, thus we set here $\alpha = 0$, and obtain the following equations for $U(u)$ and $V(v,t)$:

$$\partial_t V - 2D\,\partial_v\,\partial_v V = 0$$

$$2D\,\partial_u\,\partial_u U - \partial_u \tilde{F}\,\partial_u U - U\,\partial_u\,\partial_u \tilde{F} = 0$$

Thus, the dynamics of the V component is described by the diffusion equation with diffusion coefficient $2 \cdot D$. Its solution is the normal distribution with variance that grows linearly in time [2,4]:

$$V(v,t) = \frac{1}{\sqrt{8\pi D t}} e^{-\frac{v^2}{8Dt}}.$$

The solution for the U component is the Boltzmann distribution with potential F and effective 'temperature' D [2]:

$$U(u) = \frac{e^{F(u)/D}}{\int e^{F(u)/D}}.$$

This steady state solution manifests the balance between selection and mutation by the ratio F/D. A low (high) F/D ratio results in a wide (narrow) distribution around the line of maximal fitness. In summary, along characteristics perpendicular to the optimal fitness line, the

population density decays with the distance from the maximal fitness line; along characteristics parallel to this line the population diffuses freely. Our conclusions apply to any potential of the form $\tilde{F}(u) = g[(u-1)^2]$. In the simulations, we used a specific function (see below).

This behavior is demonstrated in Figs. S2-S4, showing the distributions of $x + y$ and $x - y$ and the time-dependence of their moments obtained in simulations with sum-mutations.

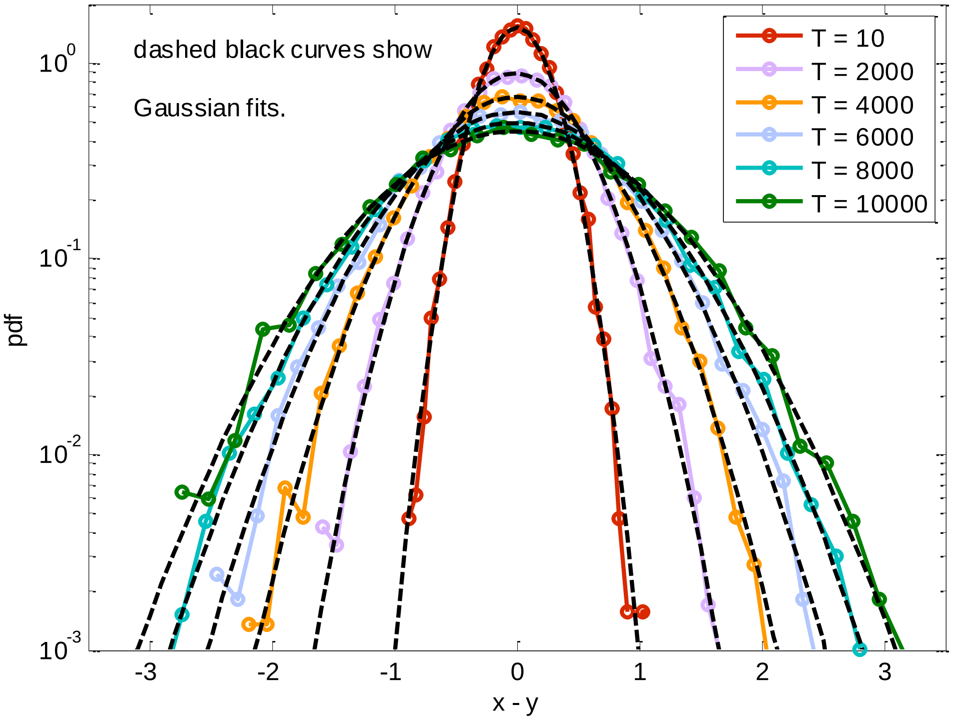

Fig S2: $\mathbf{x-y}$ values with sum mutations are normally distributed in the $\mathbf{x}$, $\mathbf{y}$ problem - simulation results. Colored solid curves illustrate distributions of $\mathbf{x-y}$ values at different time points. Dashed black curves show best fit (in terms of maximal likelihood) to Gaussian – with excellent agreement. Time $\boldsymbol{T}$ is given in number of generations in the simulation. Simulation parameters: $\boldsymbol{\beta}$ - selection with coefficient $\boldsymbol{\beta = 5}$; mutations were normally distributed N(0,0.05). Population was initiated at the origin. Results based on 10,000 points.

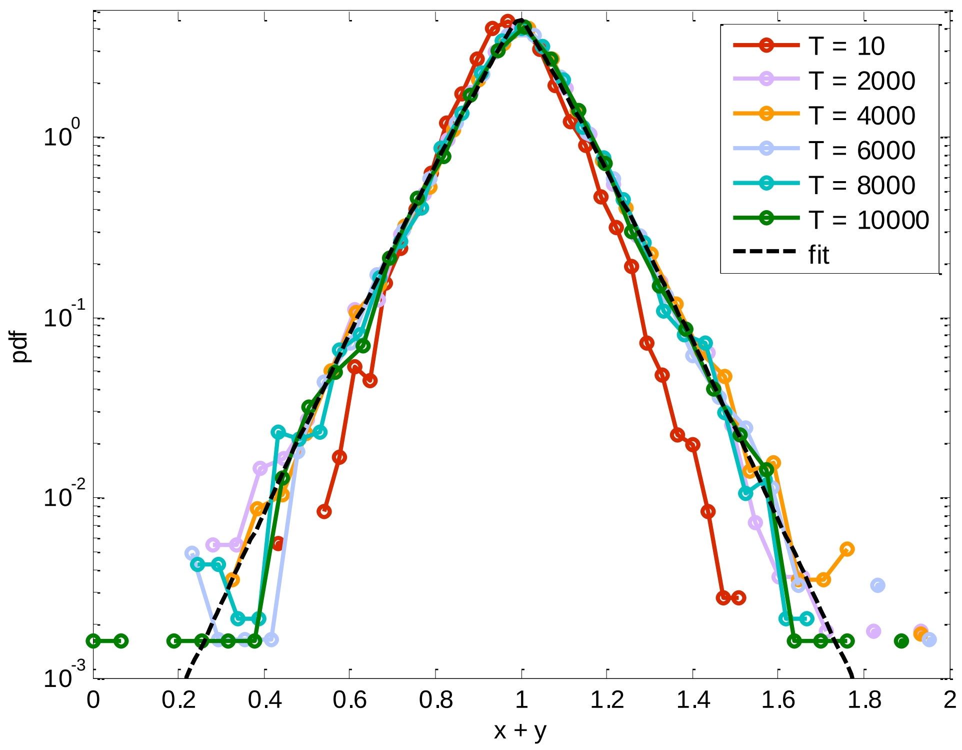

Fig S3: $\mathbf{x+y}$ distributions with sum mutations converge to a stretched exponential distribution - simulation results. Colored solid curves illustrate distributions of $\mathbf{x+y}$ values at different time points. Dashed black curve is fit to an effective potential $\mathbf{a \cdot e^{-b|x+y-1|^c}}$ with a=4.6, b=11.1, c=1. All simulation points from $\mathbf{T = 5000}$ were pooled together to produce the fit. Results pertain to the same simulations as in the previous figure.

Dynamics under product-rule mutation near the sparse solution (0,1)

We now turn to product-rule mutations. By using the FP equation to describe mutations we assume that they are localized. This is a reasonable assumption for sum-mutations, but not for product-mutations, which can span a broad distribution of outcomes (see the “scaling effect” in the next section). To remedy this, we transform the equation to the logarithm of the original variables. Product is then transformed into sum, and the locality assumption of mutations is justified again.

If the product-rule mutation scheme is not symmetric, (in our case it is biased towards decreasing parameters values) a drift term should be added which is linear in the first derivative of the population density. Note that by the nature of the transformation to log-space, a product-rule that is biased towards decreasing values translates in log-space to a biased random walk towards $-\infty$. Using the transformation:

$$x = e^{G_x}$$

$$y = e^{G_y}$$

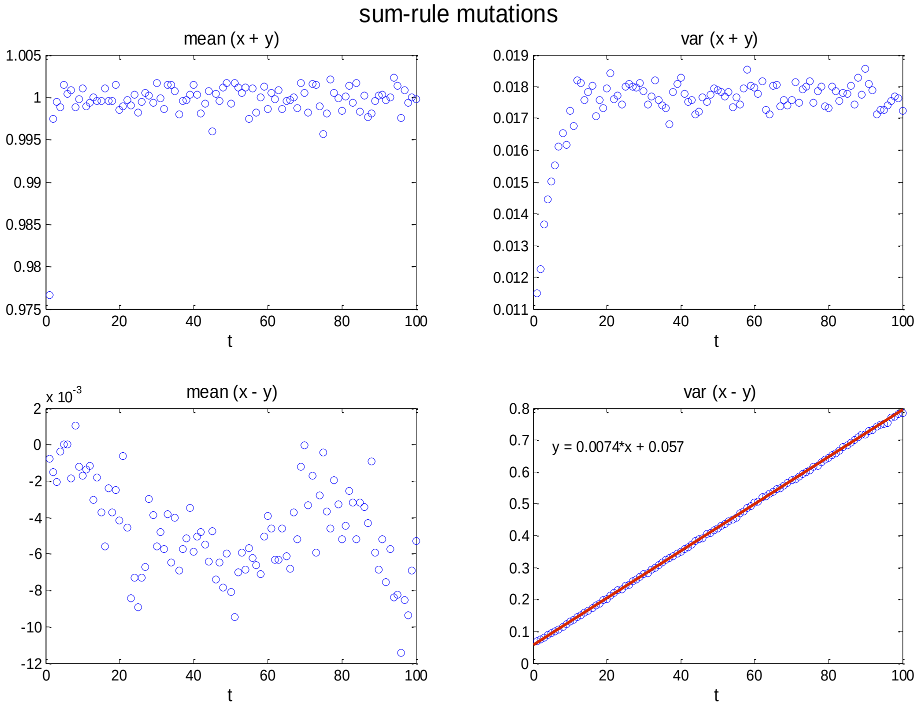

Fig S4: Time dependence of the mean and variance of $\mathbf{x+y}$ and $\mathbf{x-y}$ in sum-rule simulations support our decomposition into two functional axes. Selection rapidly drives the $\mathbf{x+y}$ component to converge to the line $\mathbf{x+y=1}$ with constant variance. In contrast, the $\mathbf{x-y}$ component freely diffuses, exhibiting a variance that grows linearly in time, in accordance with the analytical solution. The red line is a linear fit. Time $\boldsymbol{t}$ is given in thousands of generations in the simulation.

where $\mathrm{G_x}$ and $\mathrm{G_y}$ are the log-transformed variables of x and y respectively. In equation:

$$\partial_t P(\mathrm{G_x},\mathrm{G_y},t) = D\,\nabla^2 P(\mathrm{G_x},\mathrm{G_y},t) - s\cdot\nabla P(\mathrm{G_x},\mathrm{G_y},t) - \nabla\cdot\Big(P(\mathrm{G_x},\mathrm{G_y},t)\nabla F(\mathrm{G_x},\mathrm{G_y})\Big).$$

Here $s<0$ is the velocity of the drift towards $-\infty$. As with the symmetric case, this equation can be derived from the master equation under the assumption that the transition probability depends not only on the fitness difference between neighboring grid points, but also on the biased random walk probability to decrease. For example, in 1D the probability p to move to the left (towards -∞) is larger than ½. The value of s in 1D is given by $(1-2p)\ \Delta\mathrm{G_x}/\Delta t$. Note that in the symmetric case $=1/2$, and s naturally vanishes.

The fitness function F (the potential in the FP equation) is:

$$\mathrm{F}(\mathrm{G_x},\mathrm{G_y}) = -\left(\mathrm{e}^{\mathrm{G_x}}+\mathrm{e}^{\mathrm{G_y}}-1\right)^2.$$

We proceed by concentrating on one of the two sparse solutions to our problem (0,1). The sparse solution is obtained in the limit $(\mathrm{G_x},\mathrm{G_y})\to(-\infty,0)$. The asymptotic form of the fitness in this limit again depends only on one of the variables

$$F\left(G_x, G_y\right) \to -\left(e^{G_y} - 1\right)^2.$$

With this in mind, the FP equation in log-transformed variables becomes:

$$\partial_t P = D\,\partial_{G_x}\partial_{G_x}P + D\,\partial_{G_y}\partial_{G_y}P - s\,\partial_{G_x}P - s\,\partial_{G_y}P - \partial_{G_y}(P\,\partial_{G_y}F(G_y)).$$

Using similar reasoning as in the sum-mutation case, we substitute $P = X(G_x, t)Y(G_y)$:

$$\partial_t X - D\ \partial_{G_x}\partial_{G_x}X + s\,\partial_{G_x}X = 0$$

$$D\,\partial_{G_y}\partial_{G_y}Y - \partial_{G_y}\left(Y\,\partial_{G_y}F\left(G_y\right)\right) - s\,\partial_{G_y}Y = 0$$

Similarly, the solution for the Y component is:

$$Y\left(G_y\right) = \frac{e^{(F(G_y)+s\,G_y)\,/D}}{\int e^{(F(G_y)+s\,G_y)\,/D}} \ \to\ \widetilde{Y}(y) = \frac{y^{-s/D}.e^{\widetilde{F}(y)/D}}{\int y^{-s/D}.e^{\widetilde{F}(y)/D}},$$

where the main difference is that the distribution is not necessarily symmetric. Here the population is concentrated around $e^{G_y} = y = 1$ with variance determined by D and skewness determined by the ratio $-s/D$. Again note that in limit $s = 0$ the distribution is symmetric. The solution for the X component in log-space is again a Gaussian with variance that grows linearly in time, and a mean that moves to the left with velocity s:

$$X(G_x, t) = \frac{1}{\sqrt{4\pi Dt}} e^{-\frac{(G_x+s\,t)^2}{4Dt}}.$$

Transforming to the original variables we found:

$$X(G_x, t)dG_x = \widetilde{X}(\log(x)\,, t)\,\frac{dx}{x}$$

or

$$\widetilde{X}(t, x)dx = \frac{1}{x\sqrt{4\pi Dt}} e^{-\frac{[\log(x)+s\,t]^2}{4Dt}}dx.$$

This is a lognormal distribution [5] with mode (most probable value) that converges to zero like $\exp((-s - 2D\,)t)$, but mean that diverges like $\exp\left((D - s)t\right)$, note that $s < 0$. For large t the leading term in the asymptotic expansion of this distribution goes like $\sim \frac{x^{\frac{-s}{2D}-1}}{\sqrt{4\pi D}} \frac{e^{-\frac{s^2}{4D}t}}{\sqrt{t}}$. This result with $s = 0$, agrees with the result in [6] that the product of infinitely many random variables converges to the log-normal distribution.

Simulation results demonstrating this behavior are shown in Figs. S5-S7.

In summary, we have shown by means of analytical solution and simulations that in the 2-variable toy model evolutionary dynamics with biased product-rule mutations bring us to solutions in which one of the variables asymptotically approaches zero. These are the sparsest solutions possible in this problem. These sparse solutions are strongly preferred although they show no fitness advantage relative to many other solutions that are equally fit but non-sparse. In contrast, with sum-mutations there is no preference to any specific solution as long as it achieves the goal. In the main text we show simulation results of a more complex matrix-multiplication model, which exhibits a very similar behavior. Under product mutations, the solutions obtained there are those that have the maximal number of zeros that still satisfy the goal, where under sum-mutations again arbitrary solutions that satisfy the goal are obtained. The likelihood of the latter solutions to be modular is very low.

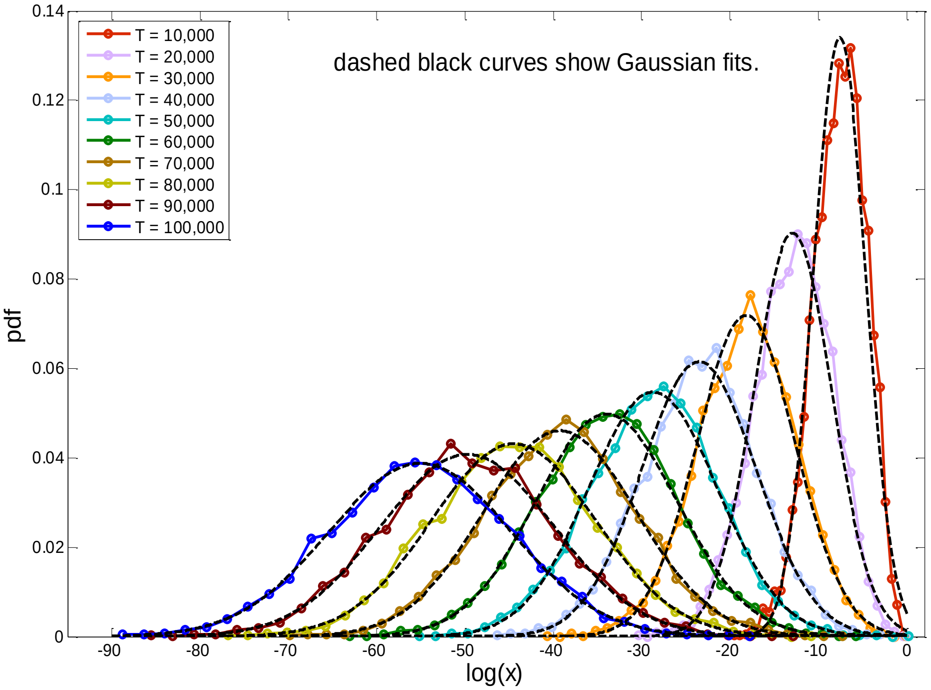

Fig S5: $\mathbf{x}$ values under product mutations are log-normally distributed and asymptotically approach zero in the $\mathbf{x}, \mathbf{y}$ problem - simulation results. Colored solid curves illustrate distributions of log(x) values at different time points. Dashed black curves show best fit (in terms of maximum likelihood) to Gaussian – with excellent agreement. Time $\boldsymbol{T}$ is given in number of generations. Simulation parameters: $\boldsymbol{\beta}$-selection with $\boldsymbol{\beta} = \mathbf{5}$, mutation normally distributed N(1,0.2). Population was initiated at the origin. In order to concentrate on one of the two sparse solutions, only simulation points with $\mathbf{0} < x < 0.5$ were considered in this analysis (roughly ~6000 points at each time point).

Stochastic simulations of toy model

To estimate the temporal behavior of population distributions in this toy model we performed repeated runs of our simulation. At each run we randomly sampled a single individual from the population at the sampled time point. This was done in order to avoid dependence between different members of the same population due to finite population size. Each run was initiated

with a different random seed, to assure independence of the distinct runs. Simulation consisted of repeated mutation-selection rounds, as described in the Methods Section of the main text. We used β-selection with $\beta = 5$. Mutations were normally distributed N(0,0.05) for sum rule mutations and N(1,0.2) for product rule mutations. Simulation was initiated with the population normally distributed around the origin $x = 0, y = 0$, with std 0.1 in both x and y axes.

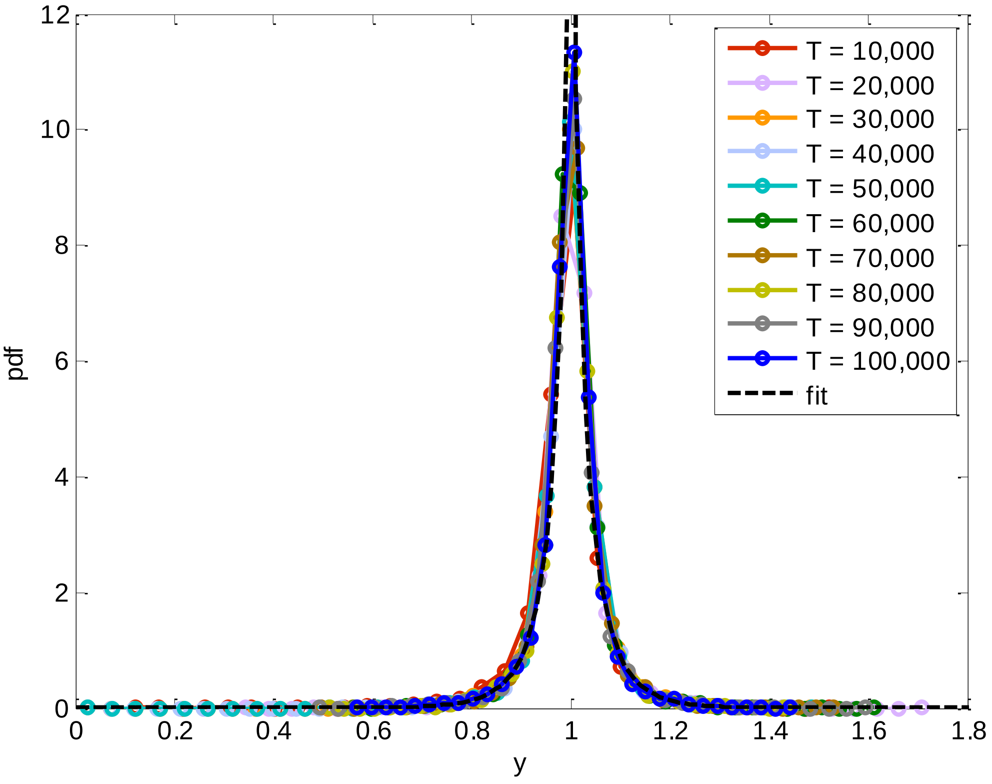

Fig S6: y values with product mutations converge to a stretched exponential distribution in the x, y problem - simulation results. Colored solid curves illustrate distributions of y values at different time points. Dashed black curve shows fit to an effective potential $a \cdot e^{-b|y-1|^c}$ with a=21.19, b=14.8, c=0.67. Time T is number of generations. Results pertain to the same simulation points as in the previous figure (i.e. the y values corresponding to $0 < x < 0.5$).

The β-selection includes fitness scaling of the form: $f_i \rightarrow \frac{e^{-\beta(x_i+y_i-1)^2}}{\sum_i e^{-\beta(x_i+y_i-1)^2}}$. However, the relation to the potential is more complicated. Thus, we fit the simulation results to an effective potential of the form $b \cdot |x + y - 1|^c$.

Under these initial conditions the product-rule mutations have equal probability to converge to either one of the two sparse solutions. Simulation results are thus a superposition of the two solutions. When relevant to the analysis, we separated simulation points. To show the distribution approaching to the (0,1) solution we selected only points with x <0.5.

To plot distributions of product-mutation simulation we used uniform binning in the log domain. Fits to Gaussian are maximum likelihood estimators under the assumption that the data is normally distributed, calculated using the Matlab function ‘normfit’.

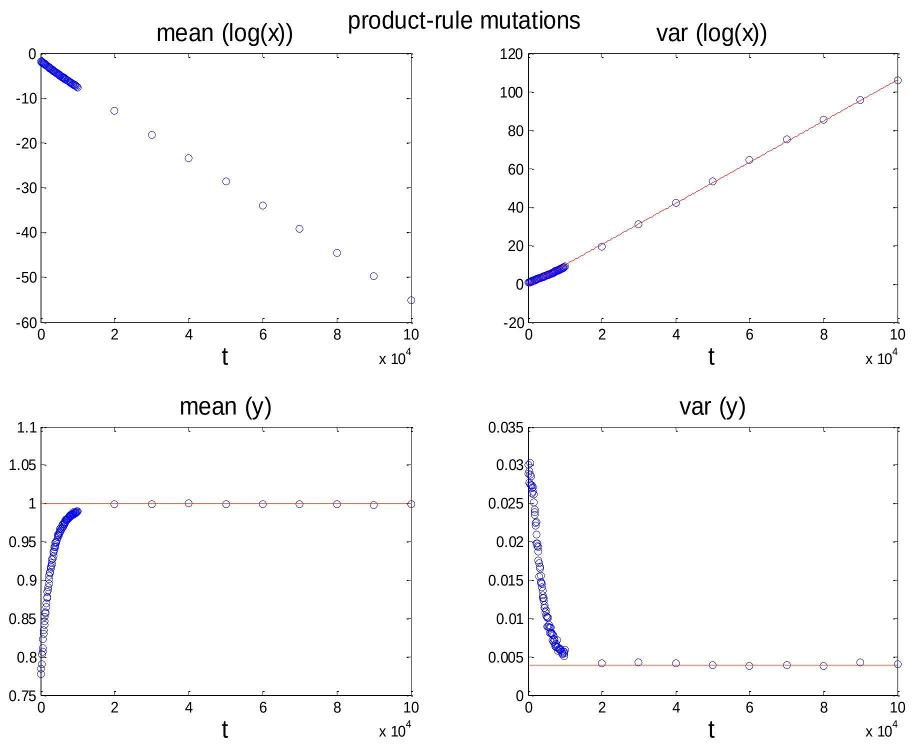

Fig S7: Time dependence of x moments under product-rule mutations agrees with log-normal distribution predicted by analytical solution – simulation results. The variance of log(x) is found to grow linearly in time and the mean of log(x) decreases linearly, as predicted by the analytical solution, assuming biased random walk in the log-space. Top row: red lines show best linear fits. In contrast, both the mean and the variance of y converge to a constant (red lines were added to guide the eye). Results pertain to the same simulations as in the previous two figures.

2. Mutation properties

“Scaling effect” – sum vs. product mutations

Product mutations have the property that the pre-mutation value scales the distribution of potential outcomes. For example: multiplying the number 0.1 will result in a narrower distribution of potential outcomes compared to the one obtained if we multiplied the number 1 by values drawn from the same distribution. This property holds for both symmetric and

asymmetric product mutations. Thus, the smaller is the pre-mutation value, the less likely it becomes to "escape" from it by mutation. Intuitively this explains why product mutations keep small interaction terms small.

Sum-mutations in contrast do not have this "scaling effect" - the distribution of mutation outcomes has the same width, regardless of the pre-mutation value. Thus product-mutations are fundamentally different from sum-mutations – see illustration in Fig. S8.

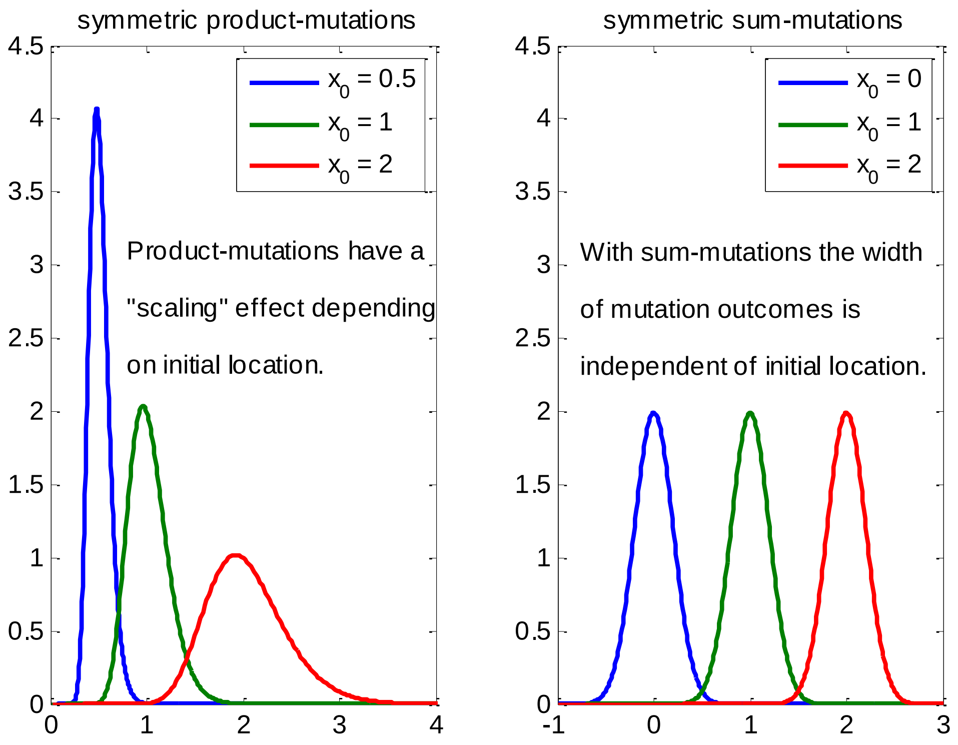

Fig S8: Product-mutations have a scaling effect, but sum-mutations do not. We compare the distribution of possible outcomes due to a single symmetric product-mutation (left) to that of a symmetric sum-mutation (right). We plot here the distribution of possible outcomes following such mutations to different pre-mutation values x_0. With sum-mutations the pre-mutation value has no effect on the width of the distribution of outcomes, and the distribution is simply relocated. In contrast, under product-mutations the smaller is the initial value, the narrower is the distribution of outcomes – that is the scaling effect. Thus the smaller are the values, the harder it is to escape. Here mutations are symmetric: either drawn from log-normal with $\mathbf{\mu}$=0, $\mathbf{\sigma}$=0.2 which have product symmetry (left) or drawn from normal distribution with $\mathbf{\mu}$=0, $\mathbf{\sigma}$=0.2 which has sum-symmetry.

Symmetry of product-mutations

The (a)symmetry of mutations determines whether the center of the mutational distribution moves or not, which is a different effect. In biological mutations both the scaling and the relocation effects exist. The discussion of mutations symmetric with respect to product is thus purely theoretical. Several works have shown that mutations are biased to decrease interactions (see citations in the main text). Thus a realistic model should capture both effects: the product

nature of mutations and their asymmetry. This discussion is meant to distinguish between the mathematical effects of these two properties, but not to argue that symmetric mutations are biologically relevant.

Which mutations are symmetric with respect to product?

To require symmetry with respect to product means that following many multiplications the geometric mean of the product will converge to 1:

$$\lim_{n\to\infty} \sqrt[n]{\prod_{i=1}^{n} x_i}=1.$$

Taking the logarithm of this equation it is equivalent to:

$$\lim_{n\to\infty} \frac{1}{n}\sum_{i=1}^{n} \log x_i = 0.$$

Then by the law of large numbers $\log x_i$ is a random value with expectation zero. Assume that $\log x_i$ is normally distributed, then x_i is log-normally distributed with parameter $\mu = 0$. To show this in an alternative way, assume that x_i is distributed with probability density f and equate the probabilities to multiply by x and by $1/x$:

$$f_x(x) \cdot \Delta x = f_x\left(\frac{1}{x}\right) \cdot \left(\frac{1}{x} - \frac{1}{x+\Delta x}\right).$$

Assume that Δx is small, we can approximate the interval $\left(\frac{1}{x} - \frac{1}{x+\Delta x}\right) \approx \frac{\Delta x}{x^2}$.

The equation becomes: $f_x(x) \cdot \Delta x = f_x\left(\frac{1}{x}\right) \cdot \frac{\Delta x}{x^2}$. Taking $f_x(x)$ to be the log-normal distribution, then:

$$\frac{e^{-(\log x - \mu)^2/2\sigma^2}}{x\sqrt{2\pi}\sigma} = \frac{x e^{-(\log(\frac{1}{x}) - \mu)^2/2\sigma^2}}{\sqrt{2\pi}\sigma} \cdot \frac{1}{x^2}$$

To satisfy this equation for every x we obtain that $\mu = 0$, regardless of the value of σ.

Throughout this work, symmetric product-mutations were drawn from this distribution. The difference between the cumulative effect of symmetric and asymmetric product mutations is illustrated in Fig. S9.

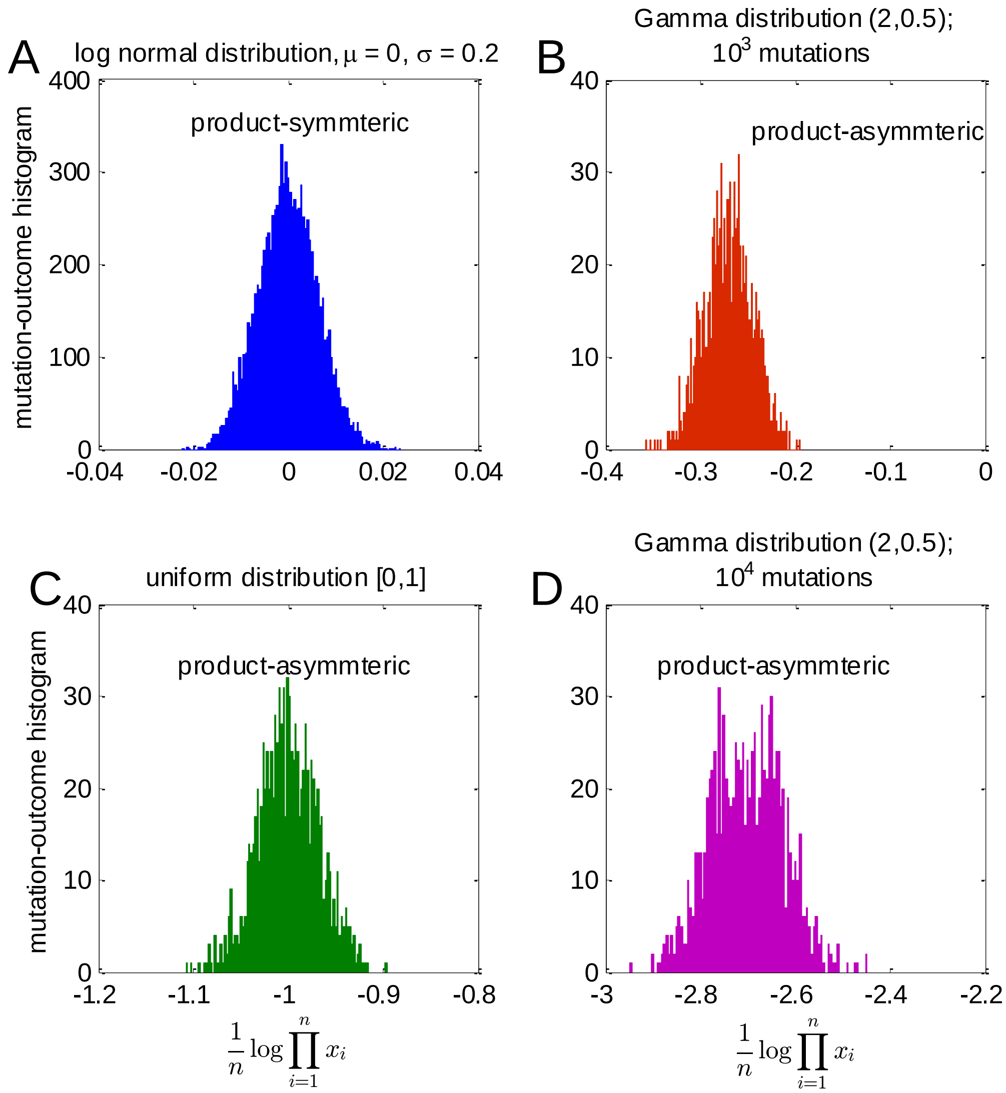

Fig S9: We demonstrate the cumulative effect of symmetric and asymmetric product mutations in the absence of selection. We assume that the initial value is 1, and multiply it by 1000 random numbers drawn from one of several distributions (detailed below). We plot here the histogram of the logarithm of cumulative mutation outcomes. (A) Product symmetric mutations were drawn from the log-normal distribution with $\boldsymbol{\mu}$=0, $\boldsymbol{\sigma}$=0.2. Because mutations are symmetric with respect to product, the histogram is concentrated around 0 in the log-space (that is around 1 in the original variables), which indicates no mutational bias. (B) Mutations drawn from Gamma distribution are biased to decrease. Thus after 1000 multiplications the histogram is concentrated around a negative value in the log-space (value<1 in the original variables). (C) The uniform distribution [0,1] is also biased to decrease. (D) The bias increases with time. Here we multiplied by 10,000 random numbers drawn from the Gamma distribution (compare to B – with only 1000 multiplications). The illustrated histograms are based on 1000 points each.

Both symmetric and asymmetric mutations lead to sparse/modular solutions:

The symmetric and asymmetric mutations differ in their effect if we had only mutations active, but not selection. In the absence of selection asymmetric mutations will bring all interactions to near-zero (given enough time), but symmetric mutations will not. If selection is active too, both symmetric and asymmetric mutations will result in sparse/modular solutions. With symmetric mutations, the reason for this is that selection breaks the symmetry. Under product-rule, mutations in the finite interval [0,1] are compensated by mutations in the infinite interval $[1,\infty)$. Thus selection for some finite goal value will always create a bias towards lower values, and thus produce a tendency to decrease, similarly to asymmetric mutations. In addition, our simulations have demonstrated that the "symmetric" state is also unstable: even a slight asymmetry is sufficient, because of the enormous number of generations in our simulations.

We illustrate below simulation results with product mutations, both symmetric and asymmetric in the $x+y=1$ problem, described in the previous section. As can be seen, both mutation types lead to sparse solutions, but with asymmetric mutations this effect is naturally stronger (Figs. S10-S11).

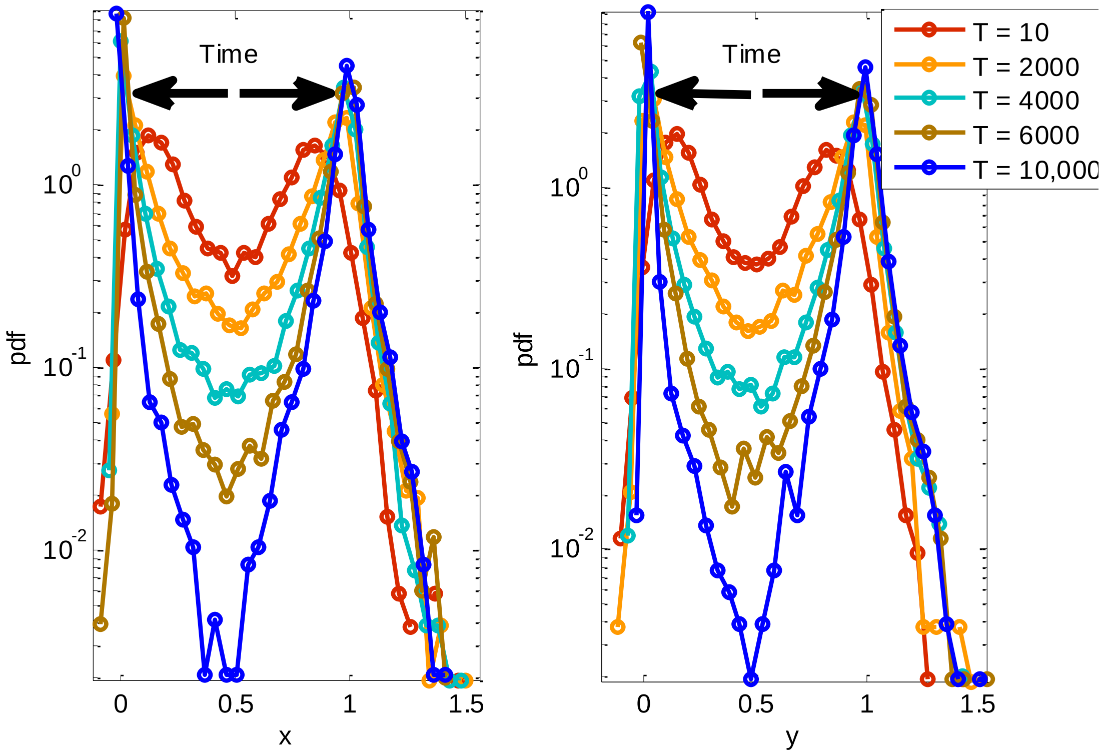

Fig S10: Evolution with asymmetric mutations in the $x+y=1$ problem. Mutations were drawn from a normal distribution N(1,0.2). The population distributions of x and y values at several time points are illustrated. As time goes on, the distributions become more and more concentrated around the sparse solutions (0,1) and (1,0). Simulation conditions are the same as in the previous section.

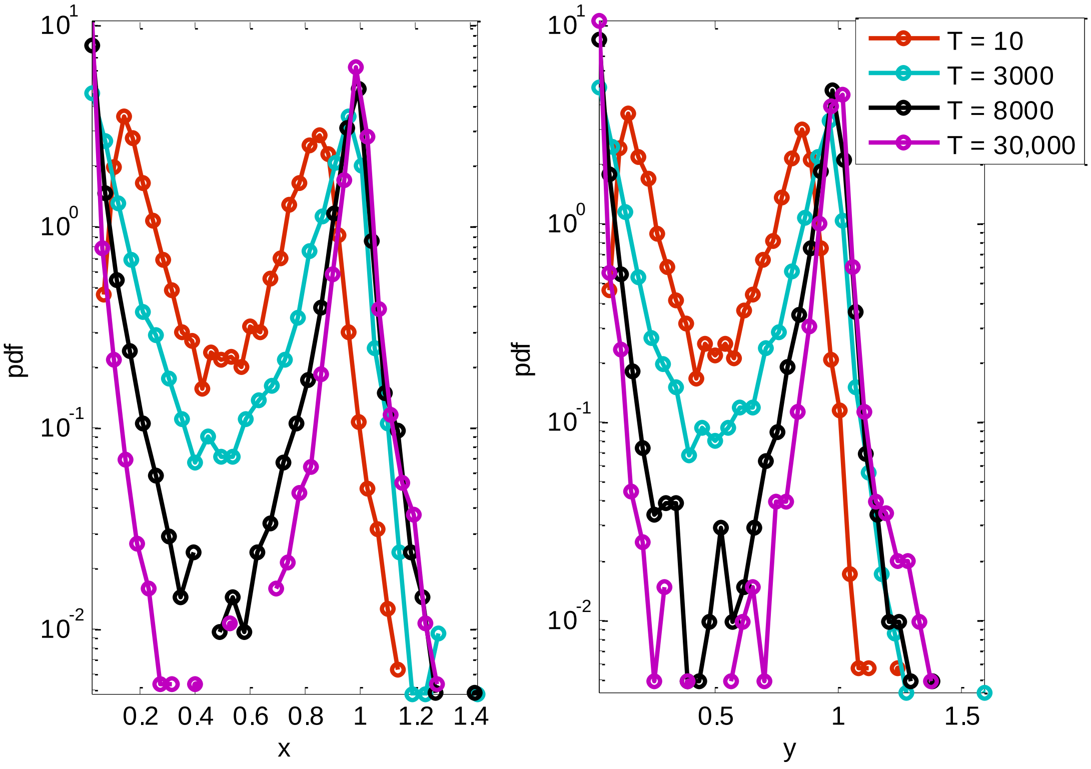

Fig S11: Evolution with product-symmetric mutations in the $x + y = 1$ problem. Mutations were drawn from a log-normal distribution LN(0,0.2). The population distributions of x and y values at several time points are illustrated. As time goes on, the distributions become more and more concentrated around the sparse solutions (0,1) and (1,0). Simulation conditions are the same as in the previous section.

3. Evolutionary simulations

Here we detail our evolutionary simulations. Simulations were written in Matlab using a standard framework [7,8]. We initialized the population of matrix pairs by drawing their $N \cdot 2D^2$ terms from a uniform distribution at either small range, i.e. $\mathcal{U}[0,\ 0.1]$ or large range $\mathcal{U}[0,1]$- where small and large are compared to the largest elements in the goal matrix (which are of order one). In other simulations we used initial values drawn from a normal distribution around zero with std 0.1 or 1. Most results shown refer to the small range, which relates to evolution of a structure with only weak initial interactions; however our conclusions apply also to the large range. Population size was set to $N = 500$.

At each generation the population was duplicated. One of the copies was kept unchanged, and elements of the other copy had a probability p to be mutated – as we explain below. We note that it is also common to keep a single copy of the population and mutate it. The former technique is less likely to lose a good solution once it is found, but its convergence is relatively

slow, whereas the latter technique is faster, but might lose beneficial solutions that have already been found (see discussion in [7] chap. 10). Fitness of all $2N$ individuals was evaluated by $F = -||AB - G||$, where $||\cdot||$ denotes the sum of squares of elements (Frobenius norm). This formula represents the Euclid distance of the matrix product from the goal [9]. The best possible fitness here is zero, achieved if $AB = G$ exactly. Otherwise, fitness values are negative. In the figures we show the absolute value of mean population fitness. The goal matrix was either diagonal $G = 2 \times I$, nearly-diagonal (diagonal matrix with small non-diagonal terms), block-diagonal or full rank with no zero elements. N individuals were then selected out of the $2N$ population of original and mutated ones, based on their fitness. This mutation–selection process was repeated again and again until the simulation stopping condition was satisfied (usually when mean population fitness was less than 0.01 from the optimum).

Mutation: We tested point mutations in our simulation and assumed statistical independence between mutations at different elements. We kept mutation rate such that on average 10% of the population members were mutated at each generation, so the element-wise mutation rate p for matrices of dimension D was at most $\frac{0.1}{2D^2}$. This relatively low mutation rate enables beneficial mutants to reproduce on average at least 10 times before they are mutated again. In simulations where we compared dependence on matrix dimension (Fig. 5) we used the same mutation rate at all dimensions, generally the one that pertains to the highest dimension used in the simulation.

We randomly picked the matrix elements (in both A and B) that would be mutated. Mutation values were drawn from a Gaussian (or log-normal or Gamma) distribution. For sum-rule mutation, this random number was added to the mutated matrix value: $A_{ij} \to A_{ij} + \mathcal{N}(0, \sigma)$ or $B_{ij} \to B_{ij} + \mathcal{N}(0, \sigma)$, and for product- rule, the mutated matrix element was multiplied by the random number: $A_{ij} \to A_{ij} \cdot \mathcal{N}(\mu, \sigma)$ or $B_{ij} \to B_{ij} \cdot \mathcal{N}(\mu, \sigma)$. Mean mutation value μ was usually taken as 1, however we also tested other values of μ (both larger and smaller than 1) and results remained qualitatively similar, only the time-scales changed.

When we tested mutations which are symmetric with respect to product we took the log-normal distribution with $\mu = 0$.

We also tested the dependence on the mutation size σ, using $\sigma = 0.01 - 3$, and found similar results. In most simulation results shown here we used $\sigma = 0.1$ (unless stated otherwise). Fitness convergence crucially depends on the mutation frequency and size, as demonstrated in our sensitivity test. Grossly speaking, a high mutation rate can speed up evolution at the beginning of the simulation, but can later on preclude slightly better mutants from taking over, because they are mutated again before they reproduce sufficiently. There is also a similar trade-off with mutation size: large mutations can speed evolution at the beginning, but at the final stages the mutation size limits the precision with which the goal can be approached.

Selection methods: We tested 3 different selection methods; all gave qualitatively very similar results with only difference in time scales. Most results presented here were obtained with

tournament selection (see [7] chap. 9): N sets, each containing s population members, were uniformly drawn with repetitions. The best individual at each set was then selected to be at the population next generation. This mimics the fact that an individual needs to outperform only others at its close vicinity, rather than the whole population. The parameter s can be used to tune the selection intensity (the larger it is, the stronger is the selection). In our simulations we set $s = 4$.

Another selection method tested is "truncation-selection" or "elitism". Here population members were ranked by their fitness. The best half of members were selected and duplicated. We note that both methods are based on the fitness rank, rather than on its exact value, making fitness scaling unnecessary. Both methods gave very similar results.

The third method used was proportionate reproduction with Boltzmann-like scaling [10–12]: here the relative fitness was computed as $\tilde{F_i} = e^{\beta F_i} / \sum_j e^{\beta F_j}$. Evidently $\sum_j \tilde{F_i} = 1$, so that $\tilde{F_i}$ is the probability of the i-th individual to be selected. The parameter β determines the selection strength, where at one extreme if $\beta = 0$, all individuals are equally probable to be selected and at the other extreme if $\beta = \infty$, the best individual is selected with probability 1, while all others have probability zero to be selected. To implement selection we then exploited the "roulette-wheel" algorithm [7,8] where a section of the interval [0,1] equal to $\tilde{F_i}$ was assigned to the i-th individual. N Random numbers were then uniformly drawn from the interval [0,1]. The individuals whose sections contained such numbers were then selected (with repetitions).

For a comparative test of the dependence of fitness achieved and the time needed to reach it on selection and mutation parameters see sensitivity test below.

If selection is too weak (e.g. $\beta = 0.1$ in Boltzmann-like selection) sparse structures are obtained, but their fitness is far from optimal. If the fraction of individuals mutated at each generation is too high (e.g. every individual has on average one mutation per generation), then again the solutions obtained are bounded away from the optimum, because high fitness individuals are likely to suffer from additional deleterious mutation before they reproduce sufficiently.

4. Evolutionary simulation parameter sensitivity test

Here we show in Fig. S12 the dependence on mutation size and selection intensity β of the evolutionary simulation with the Boltzmann-like selection scheme. In this test we let the simulation solve a 1-D problem for a fixed number of generations (=800), with a single repeat for each parameter combination. We tested 6 different values of β (0.1-20) and 5 different values of the mutation size σ (0.01-0.5). Here we plot either the mean population fitness (top row: **A** and **B**) or the best fitness obtained within the population (bottom row: **C** and **D**), reached within this fixed number of generations. In the left panels (**A** and **C**) each curve illustrates the dependence on β for a fixed mutation size, and the right panels (**B** and **D**) show the dependence

on mutation size where each curve was obtained for a different values of β. Curves in both left and right panels were created by the same simulation results.

Alternatively, we tested how the time to reach a desired fitness (0.01 from the optimum) depends on these parameters in a 3-D problem. The number of generations was limited to 500,000 and some parameter combinations failed to reach the required fitness by that time. Similarly, we show in Fig. S13 the dependence on β for fixed mutation size (**A**) or dependence on mutation size for fixed β (**B**).

Based on these tests we chose to set the mutation size $\sigma = 0.1$ and the selection intensity $\beta = 10$ (Boltzmann-like) or $s = 4$ (tournament).

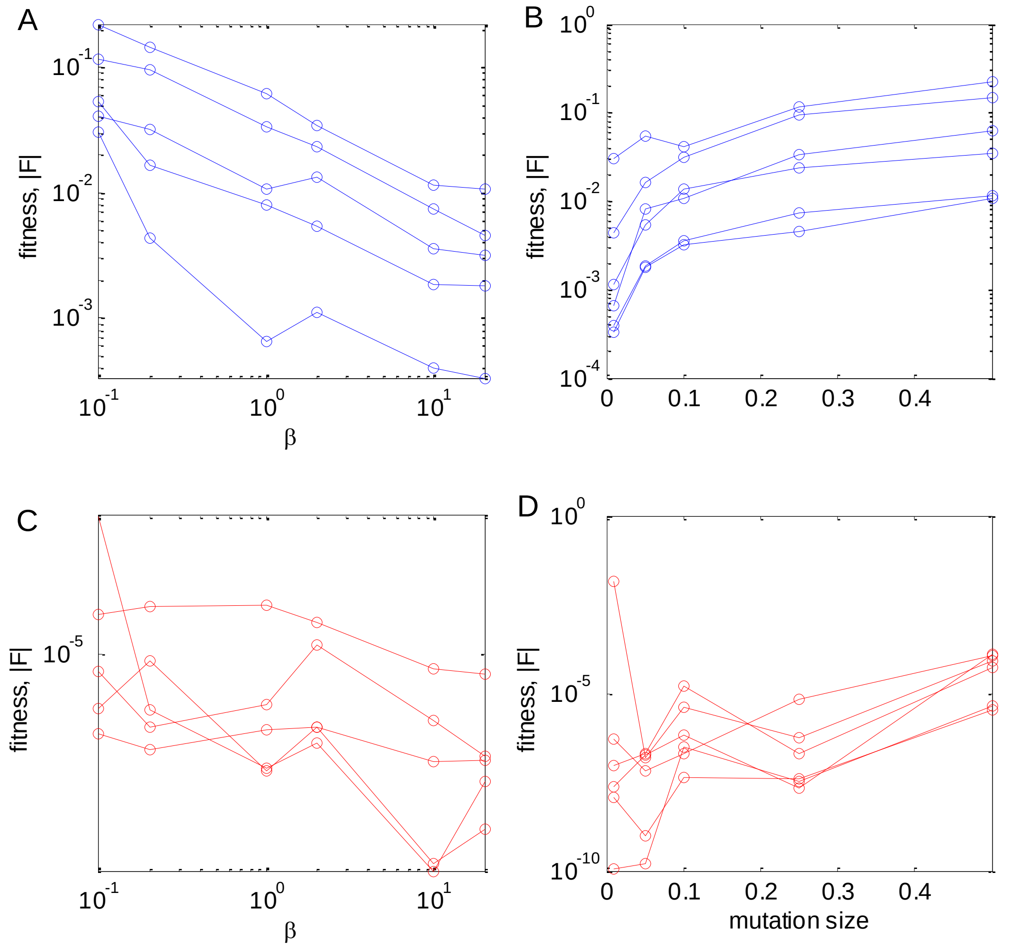

Fig S12: Dependence of the achieved fitness on selection strength and mutation size in a 1-D problem.

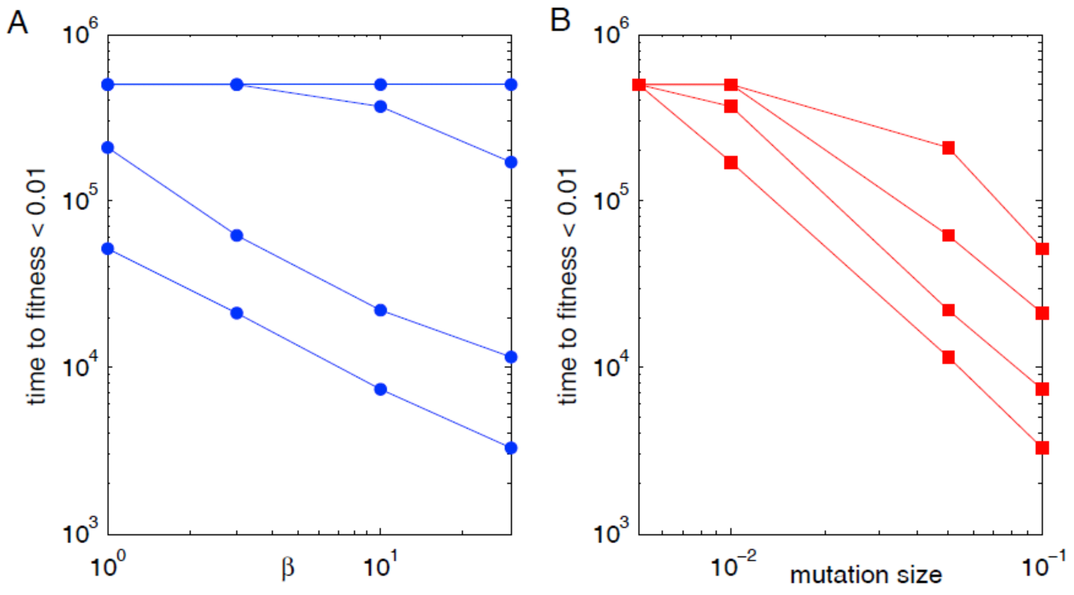

Fig S13: dependence of the time to reach a desired fitness value on selection strength and mutation size.

5. Modularity: definitions and error calculation

Definition of modularity: if the goal is diagonal, we define modularity as $M = 1 - \langle|n|\rangle / \langle|d|\rangle$ where $\langle|n|\rangle$ and $\langle|d|\rangle$ are the mean absolute value of the non-diagonal and diagonal terms respectively. At each generation, the D largest elements of each matrix (both A and B), were considered as the diagonal $\langle|d|\rangle$ and the rest $D^2 - D$ terms as the non-diagonal ones $\langle|n|\rangle$. Averages were taken over matrix elements and over the population. This technique copes with the unknown location of the dominant terms in the matrices, which could form any permutation of a diagonal matrix. Thus, $0 \leq M \leq 1$: where at the two extremes, a diagonal matrix has $M = 1$, and a matrix with equal terms has $M = 0$. Since we choose the largest elements to form the diagonal, negative values of M are not allowed. When the goal is non-diagonal, one can use standard measures for modularity such as [13] [not used in the present study].

Calculation of time to modularity: we used the following approximation for fitness value when the goal is diagonal. Assume that A and B are D-dimensional matrices consisting of 2 types of terms: diagonal terms all with size d and non-diagonal terms all with size n and that the goal is $G = g \times I_{D\times D}$. The fitness then equals:

$$-F = D[d^2 + (D-1)n^2 - g]^2 + D(D-1)(2dn + (D-2)n^2)^2.$$

We collect terms by powers of n, and obtain a constant term and terms with powers $n^{2,3,4}$. Modularity is obtained when the solution has the correct number of dominant terms appropriately located and their size is approximately $d^2 \cong g$. At the beginning of the temporal

trajectory, when non-diagonal elements are relatively large, F is dominated by the $O(n^4)$ term. When a modular structure emerges, non-diagonal elements become relatively small, and the dominant term remaining in F is $O(n^2)$. Our criterion for determining time to modularity was the time when the n^2 term first became dominant, i.e. when $F - n^2(\cdots) < n^2(\cdots)$.

Matrix permutations: For ease of presentation we permuted the A and B matrices, so that they form nearly-diagonal matrices. This applies to the cases when G is diagonal and A and B also evolve to be (nearly) diagonal. We used the same permutation for the rows of A and columns of B. Such permutation preserves the matrix product and is equivalent to simply changing the order of inputs. To find the correct permutation, we sorted each column of A in descending order. Then the first row in the sorted matrix had the D largest elements. We used the order vector of this first row (i.e. indices of rows where these elements were located in the original A) as the required permutation.

Calculation of error bars in time dependence on $\boldsymbol{D}$: We repeated the simulation at each dimension either K =140 times ($D = 3 - 10$) or $K = 80$ times ($D = 11 - 15$), initializing the Matlab random seed with a different integer number each time. At each run we measured time to reach fitness within 0.01 of the optimum and time to modularity, as explained above. As these times formed a broad and highly skewed distribution, we considered their median, rather than their mean. To estimate our error in this median estimator, we used the following bootstrapping procedure. We randomly formed sets of K samples (with repetitions) of simulation results. We constructed $L = 10{,}000$ such sets, and calculated the median of each. We then calculated the standard deviation of these median values. To estimate the error in the dependence of the time on D, we randomly picked one measurement from each dimension and then calculated the best line (in terms of least squares) connecting these points. We repeated this process 10,000 times, receiving each time different parameters for the best line. Errors in line estimation presented here, represent the 5% and 95% quantiles out of the obtained distribution of line parameters.

6. LU decomposition - proofs

An LU decomposition exists for every full rank matrix [14]. In such decomposition there is a total of $\mathrm{D}^2 - \mathrm{D}$ zeros in both A and B together. Here we prove that a larger number of zeros is not possible unless G has a zero term (or is not full rank).

The $\mathrm{D}^2 - \mathrm{D}$ zeros can be partitioned between A and B in different ways: either equally (the LU decomposition, where A and B are triangular matrices), or all zeros in one of the matrices and none in the other or any other partition.

Theorem: maximal number of zeros in LU decomposition of a full rank matrix with no zero elements is $\boldsymbol{D^2 - D}$.

Lemma: Let $A_1B_1 = G = A_2B_2$ be 2 different decompositions of the goal G with different zero partitions, such that all matrices are invertible. Then, there exists an invertible transformation matrix P, such that $A_1P = A_2$ and $P^{-1}B_1 = B_2$.

Proof: Define $P = (A_1)^{-1}A_2$. Then $A_1P = A_1(A_1)^{-1}A_2 = A_2$ and $P^{-1}B_1 = (A_2)^{-1}A_1B_1 = (A_2)^{-1}G = (A_2)^{-1}A_2B_2 = B_2$.

Q.E.D

If a transformation exists between all pairs of decompositions, specifically we can choose A_2B_2 in which A is full and B is diagonal, i.e. all $D^2 - D$ zeros are in B. Now let's check what happens if we try to add one more zero. Then, because B is diagonal, $G_{ij} = \sum_k A_{ik}B_{kj} = A_{ij}B_{jj}$, $\forall i, j$. Without loss of generality we set $A_{ij} = 0$, then essentially $G_{ij} = 0$, so G is not a general matrix.

Alternatively if we set $B_{jj} = 0$, we will obtain that the j-th column of G is all zeros – hence G is not full rank.

Q.E.D

Theorem: If $\boldsymbol{G}$ is full rank but has $\boldsymbol{k}$ zeros, the maximal number of zeros in LU decomposition is $\boldsymbol{D^2 - D + k}$.

As stated above, for a general full rank G a decomposition in which A is full and B is diagonal, (i.e. there is a total of $D^2 - D$ zeros) is possible.

Now assume without loss of generality that $G_{ij} = 0$. Since B is diagonal $G_{ij} = A_{ij}B_{jj}$, so that A_{ij} must be zero too ($B_{jj} \neq 0$ because otherwise a full column in G equals zero and then G is not full rank). Consequently, for every zero in G, we obtain exactly one additional zero in A, which proves our claim that for G with k zeros, we obtain a decomposition with exactly $D^2 - D + k$ zeros.

Due to the lemma above, these zeros can be split in different ways between A and B.

Q.E.D

7. Nearly modular G - supplementary figure

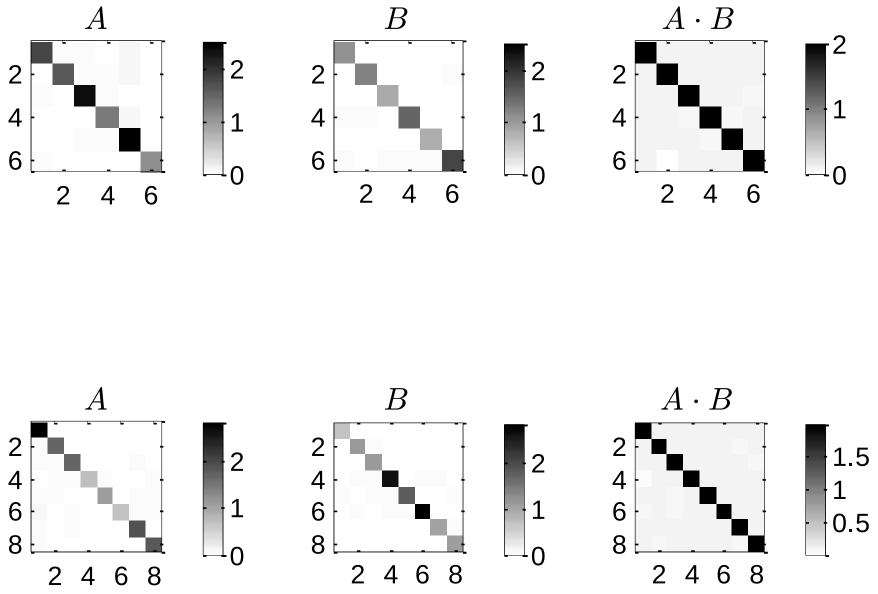

Fig S14: If the goal G is nearly diagonal, the evolutionary simulation with product-rule mutations reaches solutions in which A and B are nearly-diagonal too. We set G to be a matrix with values of 2 on its diagonal and 0.1 in its all non-diagonal terms. Here we show two examples of solutions obtained for $D = 6$ (top row) and $D = 8$ (bottom row). Numerical values are represented by color code when white represents zero. Matrices were permuted to form the most diagonal form (see above).

8. Mutation sign and distribution – supplementary figure

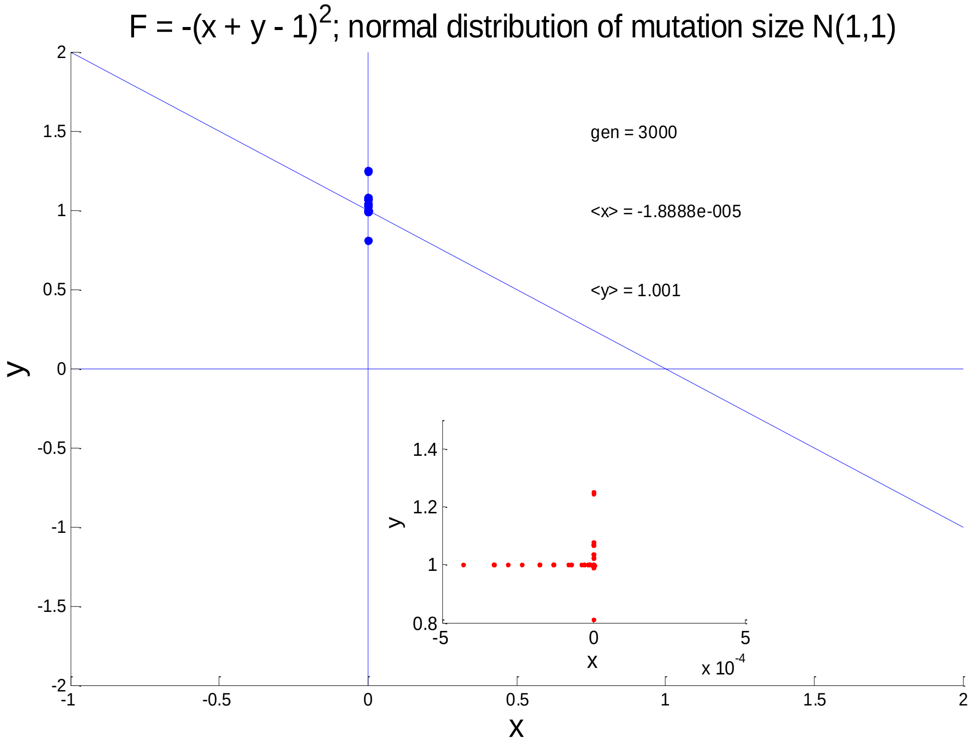

Fig S15: Broad distribution of mutation values allows for negative as well as positive matrix values. Here we show the distribution of solutions to the x, y problem with product mutations normally distributed N(1,1). The solutions concentrate near the modular point (0,1). Inset demonstrates that the x values are in fact negative in this case. Simulation was run for 3000 generations. Mean x and y values are written on top of the graph.

9. Block diagonal goal – supplementary figure

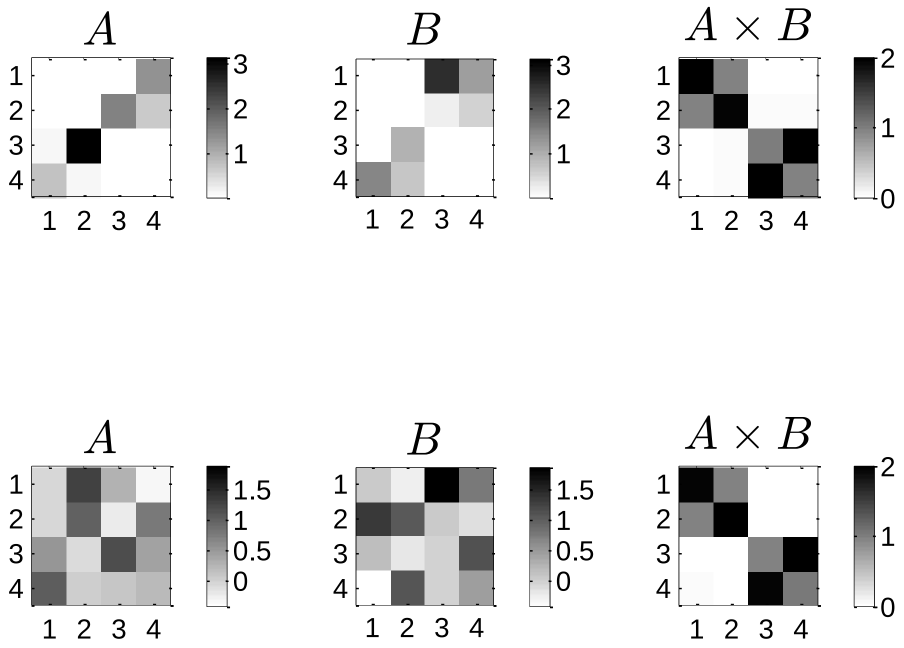

Fig S16: Comparison of product-rule vs. sum-rule mutations over a block-diagonal goal. The goal matrix here was the same block-diagonal goal as in Fig 2C (main text). Here we compare the different solutions obtained with product mutations (top row) to those obtained under sum-mutations (bottom row) with such a goal. Under product mutations, each block of the goal matrix is decomposed into a product of two triangular matrices – as happens for a general goal matrix. Under sum-mutations, we obtain non-modular solutions, as we did for diagonal goal matrices (compare to Fig.3 – main text).

References

1. Tsimring LS, Levine H, Kessler DA (1996) RNA virus evolution via a fitness-space model. Physical review letters 76: 4440–4443.

2. Gardiner C (2004) Handbook of Stochastic Methods: for Physics, Chemistry and the Natural Sciences. 3rd ed. Springer. 415 p.

3. Van Kampen NG (2007) Stochastic Processes in Physics and Chemistry, Third Edition. 3rd ed. North Holland. 464 p.

4. Metzler R, Klafter J (2000) The random walk's guide to anomalous diffusion: a fractional dynamics approach. Physics Reports 339: 1–77.

5. Limpert E, Stahel WA, Abbt M (2001) Log-normal distributions across the sciences: keys and clues. BioScience 51: 341–352.

6. Redner S (1990) Random multiplicative processes: An elementary tutorial. American Journal of Physics 58: 267.

7. Spall JC (2003) Introduction to Stochastic Search and Optimization: Estimation, Simulation and Control. Wiley-Blackwell. 618 p.

8. Goldberg DE (1989) Genetic Algorithms in Search, Optimization, and Machine Learning. Addison-Wesley.

9. Fisher RA (1930) The Genetical Theory of Natural Selection. 1st ed. Bennett JH, editor Oxford University Press, USA. 318 p.

10. Burda Z, Krzywicki A, Martin OC, Zagorski M (2010) Distribution of essential interactions in model gene regulatory networks under mutation-selection balance. Phys Rev E 82: 011908. doi:10.1103/PhysRevE.82.011908.

11. Lampert A, Tlusty T (2009) Mutability as an altruistic trait in finite asexual populations. Journal of Theoretical Biology 261: 414–422. doi:10.1016/j.jtbi.2009.08.027.

12. Lipson H, Pollack JB, Suh NP (2007) On the origin of modular variation. Evolution 56: 1549–1556. doi:10.1111/j.0014-3820.2002.tb01466.x.

13. Newman MEJ (2006) Modularity and community structure in networks. Proceedings of the National Academy of Sciences 103: 8577–8582.

14. Cormen TH, Leiserson CE, Rivest RL, Stein C (2001) Introduction To Algorithms. MIT Press. 1216 p.
